# Bisphenol A and Reproductive Health: Update of Experimental and Human Evidence, 2007–2013

**DOI:** 10.1289/ehp.1307728

**Published:** 2014-06-04

**Authors:** Jackye Peretz, Lisa Vrooman, William A. Ricke, Patricia A. Hunt, Shelley Ehrlich, Russ Hauser, Vasantha Padmanabhan, Hugh S. Taylor, Shanna H. Swan, Catherine A. VandeVoort, Jodi A. Flaws

**Affiliations:** 1 Comparative Biosciences, University of Illinois at Urbana-Champaign, Urbana, Illinois, USA; 2 School of Molecular Biosciences, Washington State University, Pullman, Washington, USA; 3 Department of Urology, University of Wisconsin Carbone Cancer Center, Madison, Wisconsin, USA; 4 Division of Biostatistics and Epidemiology, Cincinnati Children’s Hospital Medical Center, Department of Environmental Health, University of Cincinnati, Cincinnati, Ohio, USA; 5 Department of Environmental Health, Harvard School of Public Health, Boston, Massachusetts, USA; 6 Department of Pediatrics,; 7 Department of Obstetrics and Gynecology,; 8 Department of Molecular and Integrative Physiology, and; 9 Department of Environmental Health Sciences, University of Michigan, Ann Arbor, Michigan, USA; 10 Department of Obstetrics, Gynecology & Reproductive Sciences, Yale University, New Haven, Connecticut, USA; 11 Department of Preventive Medicine, Icahn School of Medicine at Mount Sinai, New York, New York, USA; 12 Department of Obstetrics and Gynecology, and; 13 California National Primate Research Center, University of California, Davis, Davis, California USA

## Abstract

Background: In 2007, an expert panel reviewed associations between bisphenol A (BPA) exposure and reproductive health outcomes. Since then, new studies have been conducted on the impact of BPA on reproduction.

Objective: In this review, we summarize data obtained since 2007, focusing on *a*) findings from human and animal studies, *b*) the effects of BPA on a variety of reproductive end points, and *c*) mechanisms of BPA action.

Methods: We reviewed the literature published from 2007 to 2013 using a PubMed search based on keywords related to BPA and male and female reproduction.

Discussion: Because BPA has been reported to affect the onset of meiosis in both animal and *in vitro* models, interfere with germ cell nest breakdown in animal models, accelerate follicle transition in several animal species, alter steroidogenesis in multiple animal models and women, and reduce oocyte quality in animal models and women undergoing *in vitro* fertilization (IVF), we consider it an ovarian toxicant. In addition, strong evidence suggests that BPA is a uterine toxicant because it impaired uterine endometrial proliferation, decreased uterine receptivity, and increased implantation failure in animal models. BPA exposure may be associated with adverse birth outcomes, hyperandrogenism, sexual dysfunction, and impaired implantation in humans, but additional studies are required to confirm these associations. Studies also suggest that BPA may be a testicular toxicant in animal models, but the data in humans are equivocal. Finally, insufficient evidence exists regarding effects of BPA on the oviduct, the placenta, and pubertal development.

Conclusion: Based on reports that BPA impacts female reproduction and has the potential to affect male reproductive systems in humans and animals, we conclude that BPA is a reproductive toxicant.

Citation: Peretz J, Vrooman L, Ricke WA, Hunt PA, Ehrlich S, Hauser R, Padmanabhan V, Taylor HS, Swan SH, VandeVoort CA, Flaws JA. 2014. Bisphenol A and reproductive health: update of experimental and human evidence, 2007–2013. Environ Health Perspect 122:775–786; http://dx.doi.org/10.1289/ehp.1307728

## Introduction

Bisphenol A (BPA) is a high production volume chemical used in a variety of common consumer products. Most notably, BPA is present in polycarbonate plastics, the epoxy resin liners of aluminum cans, and thermal receipts (Ehrlich et al. 2014). BPA is an endocrine-disrupting chemical that can act through a variety of physiological receptors, such as genomic estrogen receptors 1 and 2, membrane-bound estrogen receptors, androgen receptor, peroxisome proliferator–activated receptor γ, and thyroid hormone receptor (reviewed by Richter et al. 2007).

To address research gaps in the published BPA studies, in 2006, an organized committee sponsored by the National Institute of Environmental Health Sciences (NIEHS), National Institute of Dental Craniofacial Research (NIDCR), U.S. Environmental Protection Agency (EPA), and Commonweal reviewed the associations between BPA exposure and reproductive health outcomes and published their analyses in 2007 (vom Saal et al. 2007). This committee was organized into five topic-driven panels that evaluated the available data and developed guidelines for the conduct of future *in vivo* and *in vitro* studies that would facilitate comparisons across studies and extrapolations to human health outcomes. Overall, the panels recommended the use of oral and dietary exposure routes, doses of BPA similar to human exposure levels, and further evaluations of BPA concentrations in animal and human tissues and fluids with the aim of using doses that would result in human-relevant serum concentrations of unconjugated BPA in studies with experimental animals.

With respect to reproductive effects, the subpanel evaluating *in vivo* animal studies found contradictory results among studies but concluded that, based on the available evidence, *a*) they were confident that BPA impacts the male reproductive system, and *b*) they thought it likely—although it required confirmation—that both developmental and adult exposure affect the female reproductive system (Richter et al. 2007). At the time, the prostate was the most extensively studied reproductive tissue, with both developmental and adult exposures reported to increase prostate size [reviewed by Richter et al. (2007) and vom Saal et al. (2007)]. However, not all studies reported effects, and controversy remained regarding the doses necessary to elicit effects. In females, effects on the oocyte, developing reproductive tract, and timing of sexual maturation were available, but many remained largely unconfirmed [reviewed by Richter et al. (2007) and vom Saal et al. (2007)]. Little epidemiological information was available, and the few published studies were limited in their design, restricting the strength of the conclusions that could be drawn about the reproductive hazards of BPA exposure [reviewed by Richter et al. (2007) and vom Saal et al. (2007)].

Between 2007 and 2013, new studies on the impact of BPA on the reproductive system have been conducted and are summarized in this review. Specifically, we focus on summarizing *a*) reproductive findings from human studies and how they compare with animal studies; *b*) detailed information about the experimental effects of BPA on a variety of reproductive end points, taking into account species, dose, route of exposure, and timing of exposure; and *c*) mechanisms of BPA action in the reproductive system, whenever possible. Thus, this update is a compilation of the more recent literature detailing the effects of BPA exposure on the female and male reproductive systems. We hope that the insights and conclusions of this review will be used to direct future studies and be used in developing consensus statements about the effects of BPA on the reproductive system based on the state of the evidence.

## Methods

*Search strategy*. We searched Pubmed (http://www.ncbi.nlm.nih.gov/pubmed) to identify journal articles published between 2007 and 2013 that were related to BPA and reproduction. Search terms included the following:

Ovary, follicle, oocyte, oocyte competence, meiosis, granulosa cell, theca cell, ovulation, oviduct, uterus, endometrial, stroma, implantation, pregnancy, miscarriage, spontaneous abortion, embryo, blastocyst, placenta, trophoblast, cytotrophoblast, syncytiotrophoblast, birth, birth weight, gestation, transgeneration, testes, sperm, Leydig cell, Sertoli cell, germ cell, epididymis, prostate, ventral lobe, dorsal lobe, ejaculation, fertilization, infertility, sexual dysfunction, estradiol, testosterone, androstenedione, estrone, dihydrotestosterone, dehydroepiandrosterone, or pregnenolone.

After the aforementioned search, we searched the terms “bisphenol A” and “BPA” to identify any papers that did not include the other selected search terms or term variants.

*Article inclusion criteria*. Journal articles found using the search terms and in the selected time frame were considered for inclusion. All human and experimental animal *in vivo* studies were included. *In vitro* studies were included if there was a clear mechanism for an effect or if the studies supported *in vivo* or human findings. Journal articles were not omitted due to a paucity of research in any category.

*Dose designation*. Similar to the 2007 expert panel (vom Saal et al. 2007) and to Vandenberg et al. (2013), we defined “low dose” as a BPA dose of ≤ 50 mg/kg/day. This is the currently accepted lowest adverse effect level (LOAEL) used by the U.S. EPA (U.S. EPA 1993). Thus, “high-dose” BPA studies are those using concentrations > 50 mg/kg/day. Throughout the text, we use the terms “low dose” and “high dose” to provide a simple description of the doses used in each study. However, specific doses used in each study are listed in the Supplemental Material.

*Exposure timing*. Here, we define BPA exposure as gestational when it occurred *in utero*, neonatal when it took place after birth but before weaning, and postnatal when it occurred any time after weaning.

*Article strength determination*. Similar to the 2007 expert panel, we considered the evidence to be strong when multiple studies in multiple species indicated a similar effect of BPA on a reproductive tissue or end point, even if concordance was not 100% across all studies, given that species and strain differences can lead to differences in dose response and magnitude of effect. We considered the evidence to be limited when some studies—but not the majority—indicated a similar effect of BPA on a reproductive tissue or end point, and/or when data from *in vitro, in vivo* animal, and human studies were discordant. Finally, we considered the evidence to be inconclusive when a limited number of studies had examined the effect of BPA on a selected reproductive tissue or end point, and/or when the studies were conducted in only one species or by *in vitro* studies alone. We recognize that *in vitro* studies have played critical roles in identifying how BPA affects specific tissues; however, because it is difficult to correlate human and animal health outcomes with *in vitro* outcomes alone, studies performed *in vitro* only were classified as inconclusive.

## Early Oogenesis and Ovarian Follicle Formation

All included studies on BPA exposure and early oogenesis and follicle formation were conducted using animal models or *in vitro* systems. These studies indicate that developmental BPA exposure has the potential to affect two stages of oogenesis: *a*) the onset of meiosis in the fetal ovary, and *b*) germ cell nest breakdown and follicle formation (see Supplemental Material, Table S1). Several reports have confirmed the original findings from studies in mice (reviewed by Richter et al. 2007) that gestational exposure affects the onset of meiosis and induces nondisjunction in meiosis in the fetal ovary, but does not induce aneuploidy. In a macaque study designed to mimic serum levels of unconjugated BPA reported in human biomonitoring studies (Vandenberg et al. 2010), Hunt et al. (2012) observed that daily low-dose BPA exposure (measured < 1 ng/mL in maternal serum) significantly disrupted synapsis and recombination between homologous chromosomes at the onset of meiosis, which was consistent with previous findings (Susiarjo et al. 2007). Three studies reported that gestational low-dose BPA exposure of mice induced changes in gene expression in germ cells and early meiocytes. BPA exposure increased expression of *Stra8* (stimulated by retinoic acid 8 homolog) and a variety of meiotic genes in C57BL/6 mice (Lawson et al. 2011). However, longer gestational BPA exposure down-regulated the expression of *Stra8*, *Dazl* (deleted in azoospermia-like), and *Nobox* (newborn ovary homeobox) in CD-1 mice (Zhang XF et al. 2012). Last, BPA exposure commencing after the onset of meiosis induced a meiotic delay at gestational day (GD) 17.5 in CD-1 females (Zhang HQ et al. 2012). Collectively, these studies provide strong evidence that BPA exposure disrupts meiosis in mice and macaques, and alters gene expression in germ cells and early meiocytes in two different strains of mice.

*In vitro* studies provide further evidence that BPA affects the onset of meiosis. Brieño-Enriquez et al. (2011, 2012) reported that BPA (1–30 μM) increased oocyte degeneration by impairing meiotic progression in cultured human fetal oocytes and that, similar to mouse studies, human fetal oocytes that progressed to prophase exhibited increased levels of recombination (MLH1 foci) and gene expression changes. BPA also increased methylation errors in differentially methylated regions of maternally imprinted genes of oocytes in cultured preantral follicles of C57/BL6xCBA/Ca mice (Trapphoff et al. 2013). Thus, the data from different mouse strains, macaques, and *in vitro* studies consistently provide strong evidence to conclude that BPA exposure has detrimental effects on the meiotic process, both at the gene expression and phenotypic levels.

Studies have also suggested that BPA interferes with germ cell nest breakdown in animal models. In neonatally exposed lambs, low-dose BPA was reported to increase the incidence of multioocyte follicles (Rivera et al. 2011). Similarly, in gestationally exposed macaques, dietary low-dose BPA exposure increased the number of oocytes present in secondary and antral follicles at birth, and continuous BPA exposure (measured < 1 ng/mL in maternal serum) increased the incidence of unenclosed oocytes (Hunt et al. 2012). Further, in gestationally exposed CD-1 mice, low-dose BPA increased the number of unenclosed oocytes, whereas it decreased the number of primordial follicles in a dose-dependent manner (Zhang HQ et al. 2012). In another study, Veiga-Lopez et al. (2013) reported that prenatal BPA exposure altered the fetal ovarian steroidogenic gene and microRNA expression that mediate gonadal differentiation and folliculogenesis in sheep. Collectively, these studies provide strong evidence that gestational BPA exposure—across multiple exposure routes, doses, and species—impairs proper germ cell nest breakdown, leading to the formation of multioocyte follicles. The presence of multioocyte follicles is of concern because they are considered a pathologic condition that may lead to ovulatory problems (Iguchi et al. 1990).

BPA exposure also appears to accelerate follicle transition and growth in several species. Rivera et al. (2011) reported that neonatal BPA exposure accelerated follicle transition in lambs, decreasing numbers of primordial follicles and increasing primary follicles without affecting total follicle numbers. A similar enhanced activation of follicular recruitment was observed by Rodríguez et al. (2010) in neonatally exposed Wistar rats. BPA exposure also increased cell proliferation, indicative of follicular growth, in small antral follicles in neonatally exposed lambs and Wistar rats (Rivera et al. 2011; Rodríguez et al. 2010). Taken together, the data suggest that BPA enhances the recruitment and growth of primordial and primary follicles across species. Combined with the effects on germ cell nest breakdown, there is strong evidence that BPA induces ovotoxicity by acting on developing and immature follicle stages in animals models. However, the consequences of these effects on reproductive potential and longevity are unclear. In one study, low-dose neonatal BPA exposure decreased numbers of all follicle types and increased atretic follicles in rats during adulthood (Li et al. 2014). These effects of BPA on follicles could lead to premature reproductive senescence, but this needs to be confirmed in future studies.

*In vitro* studies on the effects of BPA have focused on mature ovarian follicles. In murine preantral follicles, BPA (0.003 μM) accelerated development to antral follicles (Trapphoff et al. 2013). In murine antral follicles, exposure to BPA (440 μM) aberrantly up-regulated expression of cell cycle regulators and the proatretic and antiatretic factors *Bax* (BCL2-associated X protein), *Trp53* (tumor protein 53), and *Bcl2* (B-cell lymphoma 2), inhibiting follicle growth and inducing apoptosis (Peretz et al. 2012). Further, BPA (110–440 μM) inhibited antral follicle growth in mice (Ziv-Gal et al. 2013). These studies suggest that low-dose BPA exposure may alter follicle formation, but high-dose BPA may directly inhibit growth, cause atresia, and induce changes in gene expression in rodent antral follicles. In future studies, it will be important to validate findings from these *in vitro* studies at the *in vivo* level and determine the consequences of BPA-induced follicle toxicity on reproduction function.

## Steroidogenesis in Females

Multiple studies have investigated the association between BPA exposure and ovarian steroid hormone production in women (see Supplemental Material, Table S2). In three publications based on women undergoing *in vitro* fertilization (IVF), BPA exposure was associated with a decrease in peak serum estradiol levels prior to oocyte retrieval (Bloom et al. 2011a; Ehrlich et al. 2012a; Mok-Lin et al. 2010). A case–control study by Kandaraki et al. (2011) showed that BPA was associated with increased testosterone and androstenedione levels in women with polycystic ovary syndrome (PCOS). In a study of 60 women undergoing IVF, Ehrlich et al. (2013) found that urinary BPA concentrations were not associated with a negative linear dose–response in the expression of the steroidogenic enzyme *Cyp19* in granulosa cells collected at the time of oocyte retrieval, but instead were associated with a suggested nonmonotonic dose–response. Conversely, BPA was not associated with estradiol or testosterone levels in women in the INChianti study, a prospective, population-based study of adults living in Chianti, Italy (Galloway et al. 2010). Given the limited information on BPA exposure and ovarian steroidogenesis in women and the discrepant study results, additional studies utilizing sensitive and reliable steroid hormone and BPA assays are required to delineate whether BPA levels negatively impact reproductive hormonal patterns in women. Further, given that most existing studies on BPA exposure and steroid levels were conducted in IVF populations, it is critical to examine the association between BPA exposure and steroidogenesis in women from the general population.

Several experimental studies have examined the effect of BPA exposure on ovarian steroidogenesis in laboratory animals (see Supplemental Material, Table S1). In three rodent studies, perinatal (Xi et al. 2011) and postnatal (Fernández et al. 2010; Tan et al. 2013) low-dose BPA exposure was reported to increase serum estradiol levels. Further, in one study of Sprague-Dawley (SD) rats and one of pregnant ICR mice, low-dose BPA increased testosterone and progesterone levels (Fernández et al. 2010; Tan et al. 2013). Interestingly, Xi et al. (2011) reported that postnatal BPA exposure alone did not affect serum hormone levels in the mice. Similarly, in other studies using rats, mice, and lambs, gestational or gestational plus neonatal BPA exposure had no effect on steroidogenesis (Kobayashi et al. 2012; Mendoza-Rodríguez et al. 2011; Rivera et al. 2011; Varayoud et al. 2011). In these four studies, the doses used were lower than those in studies reporting BPA effects, indicating that BPA doses < 20 mg/kg may not increase hormone production in animal models. In contrast, another study (Berger et al. 2008) found that although BPA did not alter estradiol levels, low-dose BPA decreased progesterone levels in adult mice during early pregnancy. In addition, in adult rats, low-dose BPA (< 0.1 mg/kg/day) decreased estradiol, testosterone, *Cyp19* (aromatase), and *Star* (steroidogenic acute regulatory protein) (Lee SG et al. 2013). Further, in adult mice, low-dose BPA decreased expression of estrogen and progesterone receptors but did not alter hormone levels (Berger et al. 2010). The differences in study results may be a function of exposure times, internal doses of BPA, or species.

The results of *in vitro* studies on the effects of BPA on steroidogenesis are also equivocal. BPA exposure (44 and 440 μM) inhibited estradiol, testosterone, androstenedione, estrone, dehydroepiandrosterone, and progesterone production and decreased *StAR* and *Cyp11a1* (cytochrome P450 side-chain cleavage) expression in cultured intact murine antral follicles (Peretz et al. 2011; Ziv-Gal et al. 2013). However, in isolated rat theca-interstitial cells, BPA (0.1–100 μM) had opposite effects, increasing testosterone synthesis and *Cyp17a* (cytochrome P450 17α-hydroxylase/lyase), *Cyp11a1*, and *StAR* expression (Zhou et al. 2008). In isolated rat granulosa cells, BPA (100 μM) decreased progesterone synthesis and increased *StAR* expression (Zhou et al. 2008). In a separate study using porcine granulosa cells, 0.1 μM BPA increased estradiol levels, whereas higher doses (1 and 10 μM) decreased estradiol levels. All three concentrations of BPA resulted in decreased progesterone levels (Grasselli et al. 2010). Collectively, these studies indicate that BPA adversely affects steroidogenesis *in vitro* (with the effects dependent on BPA concentration) and that BPA’s effects on steroidogenesis vary by whether intact follicles or isolated cells are used in the cultures.

## Oocytes: Quantity, Quality, and Fertilization

A few human studies have analyzed the association between urinary BPA levels and oocyte yield, maturation, and fertilization (see Supplemental Material, Table S2). In a small prospective study of 58 infertile women and 37 male partners undergoing intracytoplasmic sperm injection or conventional IVF, Fujimoto et al. (2011) found an association between serum BPA concentrations and oocyte maturation only among Asian women, but an overall correlation between increasing serum concentrations and the developmental potential of human oocytes. In two publications from the same prospective cohort of 84 women (Mok-Lin et al. 2010) and 174 women (Ehrlich et al. 2012a) undergoing IVF, increasing urinary BPA concentration was associated with decreased numbers of retrieved oocytes, mature oocytes (MII), and normally fertilized oocytes (2PN). These results suggest that BPA is associated with impaired oocyte yield, maturation, and fertilization, adversely affecting the success of IVF treatment.

Several recent experimental results support the findings from earlier studies that suggested BPA exposure affects the resumption of meiosis in the periovulatory oocyte (reviewed by vom Saal et al. 2007) (see Supplemental Material, Table S1). Chao et al. (2012) reported that neonatal exposure to low-dose BPA inhibited germinal vesicle breakdown (GVBD) in CD-1 F_1_ hybrid female mice, confirming the previous work of Hunt et al. (2003). Conversely, low-dose BPA administered to MF-1 (C57BL/6 × CBA/Ca F_1_ hybrid) mice on postnatal days 22–28 did not reduce GVBD or polar body extrusion nor did it increase spindle aberrations (Eichenlaub-Ritter et al. 2008). Similarly, low-dose BPA given orally to 4- or 9-week-old superovulated female C57Bl/6 mice did not affect oocyte retrieval, meiotic maturation, or induce aneuploidy (Pacchierotti et al. 2008). Although the exact reasons for the subtle discrepancies among studies are unknown, there are numerous possible reasons for the variations in the window of neonatal exposure (reviewed by Hunt et al. 2009) and potential coexposure to phytoestrogens that may modulate the effects of BPA on the periovulatory oocyte (Muhlhauser et al. 2009).

In *in vitro* studies, BPA (43.8 μM) has been reported to significantly alter spindle formation, distribution of pericentriolar material at spindle poles, and induce congression failure in MF-1 mouse oocytes isolated from antral follicles (Eichenlaub-Ritter et al. 2008). In a study in follicle-enclosed oocytes from adult mice, Lenie et al. (2008) reported that BPA (30 μM) impaired spindle alignment and caused meiotic arrest after GVBD but before polar body extrusion. Importantly, a recent study of human oocytes exposed during *in vitro* maturation, with doses of BPA within the range measured in human follicular fluid (0.02, 0.2, and 20 μg/mL), found a dose-dependent increase in the incidence of meiotic arrest, disturbances in spindle formation and chromosome alignment, and spontaneous oocyte activation (Machtinger et al. 2013). Because the doses used were in the range measured in human follicular fluid, these data, together with the results of experimental studies, provide compelling evidence that BPA adversely affects the maturing oocyte.

## Polycystic Ovary Syndrome (PCOS)

Studies on the effects of BPA on PCOS are limited (see Supplemental Material, Table S2). Women with PCOS are characterized by oligo-anovulation, functional hyperandrogenism, and multifollicular ovaries (accumulation of several small-sized antral follicles), with the majority of women showing insulin resistance and excess luteinizing hormone (Hampton 2013). One case–control study of 71 women with PCOS and 100 women without PCOS (Kandaraki et al. 2011) showed an association between serum BPA levels and increased testosterone, androstenedione, and insulin resistance in PCOS (Kandaraki et al. 2011) (see Supplemental Material, Table S2). Given the limited number of studies assessing the association between BPA exposure and PCOS symptoms, more studies are needed in order to make firm conclusions about the impact of BPA exposure on PCOS or PCOS symptoms.

The ovarian phenotype in BPA-treated rodents (cystic-appearing follicles) differs from the ovarian phenotype of women with PCOS (accumulation of small antral follicles). In rodents, prenatal and neonatal exposure to low-dose BPA lead to disruption of estrous cyclicity (Adewale et al. 2009), increased testosterone production (Fernández et al. 2010), and ovarian cysts (Newbold et al. 2009) (see Supplemental Material, Table S1). High-dose BPA exposure in rodents also leads to ovarian cysts (Fernández et al. 2010) and an accumulation of large antral follicles (Adewale et al. 2009). In addition, Fernández et al. (2010) reported that BPA exposure decreased gonadotropin-releasing hormone (GnRH) levels measured from hypothalamic explants *in vitro.* Additional studies in other animal models are needed to fully understand whether BPA exposure causes PCOS or PCOS-like conditions, and to determine why outcomes differ in animal models and women.

## Oviduct

Only one study we reviewed investigated the effects of BPA on the oviduct (see Supplemental Material, Table S1). Newbold et al. (2009) observed progressive proliferative lesions in the oviducts of adult CD-1 mice prenatally exposed to low-dose BPA. Because alterations in oviduct morphology would be expected to adversely affect both fertilization and embryo transport, future studies are clearly warranted to examine the impact of BPA not only on the oviduct but also on fertilization and embryo transport.

## Uterine Morphology

No studies have reported the impact of gestational BPA exposure on uterine morphology in women because of the extreme difficulty of monitoring adult outcomes of prenatal exposure to BPA; however, animal studies suggest that gestational BPA exposure perturbs gross morphology of the uterus in the adult (see Supplemental Material, Table S3). Specifically, low-dose BPA exposure has been reported to induce benign and malignant lesions (Newbold et al. 2009; Signorile et al. 2010) and endometrial polyps in the uterus (Newbold et al. 2009) and to perturb Wölffian duct regression in prenatally exposed adult mice (Newbold et al. 2009). Further, adult hens that were exposed *in ovo* on day 4 of incubation to BPA (134 ng/kg) had decreased thickness of their tunica mucosa and density of uterine glandular structures compared with unexposed hens (Yigit and Daglioglu 2010). Together, these studies indicate that gestational BPA exposure may be potentially deleterious to uterine morphology in adult females. Because abnormalities have been identified in middle age that were not observed in young adult mice (Newbold et al. 2009), future studies should assess effects in exposed animals at different life stages.

## Uterine Endometrium

Limited data exist on BPA exposure and uterine endometrium in women. One case–control study of 69 women (Cobellis et al. 2009) suggests that serum BPA concentrations may be associated with the occurrence of endometriosis (see Supplemental Material, Table S2). In another study of 495 individuals in an operative cohort and 131 women in population cohort, Buck Louis et al. (2013) found no association between BPA exposure and endometriosis. However, that study was not originally intended to investigate BPA exposure, nor was it was appropriately powered to assess endometriosis; that study also included a lapse between sample collection and endometriosis evaluation, further confounding these data. Given the lack of studies on BPA and endometrial disorders in humans, more studies are required before making conclusions about whether BPA adversely impacts the uterine endometrium in women.

Experimental studies support the findings of Cobellis et al. (2009) (see Supplemental Material, Table S3). Signorile et al. (2010) reported that adult female Balb-c mice exposed prenatally and neonatally to low-dose BPA developed endometrial-like structures with both glands and stroma in adipose tissue surrounding the genital tract. These structures expressed *Hoxa10* (homeobox A10), a transcription factor that mediates proliferation of stromal tissue prior to implantation. Two other studies reported that BPA, at both low- and high doses, increased expression of *Hoxa10* in prenatally exposed adult CD-1 and ICR mice (Bromer et al. 2010; Hiyama et al. 2011).

Other *in vivo* studies have provided evidence that BPA impairs proliferation in the uterus. In rodents, BPA exposure over a wide range of doses, times, and routes decreased expression of uterine *Esr1* (estrogen receptor α), which may lead to inhibited endometrial proliferation in the uterine epithelium and stroma (Berger et al. 2010; Bosquiazzo et al. 2010; Bromer et al. 2010; Varayoud et al. 2008). In two separate studies of adult rats, prenatal and neonatal exposure to low-dose BPA decreased uterine epithelium proliferation in response to hormone treatments (Mendoza-Rodríguez et al. 2011; Varayoud et al. 2008). In a study in non-human primates, Aldad et al. (2011) reported that although low-dose BPA did not affect progesterone receptor expression, it dampened glandular and stromal progesterone receptor expression in response to estradiol stimulus. Low-dose BPA was also reported to impair apoptosis of the uterine epithelium during estrus in neonatally exposed adult rats (Mendoza-Rodríguez et al. 2011).

*In vitro* studies also support the hypothesis that BPA exposure adversely affects the uterus. BPA (50 or 100 μM) has been reported to significantly decrease proliferation of human endometrial stromal fibroblasts cultured for 48 hr (Aghajanova and Giudice 2011). BPA (50 μM) was also found to decrease the proliferation of cultured human endometrial endothelial cells (Bredhult et al. 2009). Further, in cultured primary heterogeneous populations of uterine cells, BPA (10 μM) significantly inhibited uterine cell contractions, increased oxytocin-related pathways, and decreased prostaglandin-related signaling after 48 hr (An et al. 2013). Taken together, the existing animal and *in vitro* studies provide strong support that BPA impairs uterine cell proliferation.

## Uterine Receptivity and Implantation

Only one study has reported on the association between BPA exposure and uterine receptivity/implantation in women (see Supplemental Material, Table S2). In a study of 137 women undergoing IVF, Ehrlich et al. (2012b) found that higher quartiles of urinary BPA concentrations were associated with increased odds of implantation failure. Given the limited information on this topic, future studies need to be conducted to determine whether BPA exposure is associated with adverse uterine receptivity/implantation outcomes in women.

Several experimental studies indicate that BPA exposure impairs uterine receptivity and implantation (see Supplemental Material, Table S3). Repeated exposure of pregnant mice to low-dose BPA during early gestation was reported to completely ablate embryo implantation (Berger et al. 2008; Xiao et al. 2011). A single exposure to high-dose BPA on GD0 or GD1, but not on GD2, was found to decrease implantation in mice (Berger et al. 2008, 2010). Neonatal exposure to low-dose BPA also decreased implantation sites in pregnant rats (Varayoud et al. 2011). Interestingly, low-dose BPA has been reported to increase preimplantation loss in unexposed female rats mated to neonatally exposed males (Salian et al. 2009a) and decrease implantation sites in females mated to adult exposed male rats (Tiwari and Vanage 2013). Further, when untreated and healthy embryos were transplanted into the uteri of low-dose-BPA–exposed mice, implantation failed (Xiao et al. 2011). Together, these studies provide strong evidence that BPA exposure, in both males and females, affects uterine receptivity in females. However, a need still exists to explore whether BPA-exposed embryos attach to the uterine epithelium and initiate implantation.

Neonatal exposure to low-dose BPA was also reported to decrease pregnancy maintenance in experimental studies (Varayoud et al. 2011). Low-dose BPA increased resorption rates in uteri of unexposed female rats mated to males that were neonatally exposed (Salian et al. 2009a), gestationally plus neonatally exposed (Salian et al. 2009b), or exposed as adults (Tiwari and Vanage 2013), suggesting that male exposure can significantly contribute to pregnancy loss. These effects were also found in F_1_ and F_2_ offspring (Salian et al. 2009a, 2009b), suggesting transgenerational effects of BPA. The potential ability of BPA to cause transgenerational effects in animal models is further supported by Hiyama et al. (2011), who observed reduced uterine weight and expanded uterine lumen in mice developmentally exposed to high-dose BPA, as well as demethylation of the *Hoxa10* gene in the F_2_ generation. Collectively, these experiments indicate that BPA exposure adversely affects implantation and pregnancy maintenance in animal models, which may be transgenerational in nature.

## Embryo Development

Few studies have examined the effects of parental BPA levels on subsequent *in vitro* embryo development in humans (see Supplemental Material, Table S2). In one prospective preconception cohort study of 174 women, total urinary BPA concentration was associated with a decreased rate of blastocyst formation (Ehrlich et al. 2012a). Further, a prospective cohort study involving 27 couples undergoing IVF treatment found that increasing urinary BPA concentrations in the male—but not the female partner—was associated with decreased odds of a high embryo fragmentation score, suggesting that the embryos were low quality for IVF treatments (Bloom et al. 2011b). Collectively, these studies indicate adverse associations of parental BPA levels on the development of early embryos *in vitro,* but further studies are required to determine whether the observed effects occur *in vivo*.

Although only two experimental studies have investigated the effects of BPA exposure on early embryo development *in vivo*, the results are intriguing. Xiao et al. (2011) reported delayed embryo development in pregnant C57BL/6 mice administered high-dose BPA from GD0.5 to 3.5 (see Supplemental Material, Table S3). A more recent study of F_1_ female C57BL/6 Cast7 mice showed that low-dose BPA exposure initiated prior to breeding and continuing through pregnancy disrupted the expression of imprinted genes in midgestation embryos and placentas (Susiarjo et al. 2013). Given the limited number of studies on the effects of BPA on embryo development *in vivo*, additional studies are needed in order to make firm conclusions about the effects of BPA on early embryos.

## Placenta

Although epidemiological studies on BPA and the placenta have not been published, one experimental study Tan et al. (2013) suggested that both low and high doses of BPA increase levels of plasma estradiol, testosterone, and corticotropin-releasing hormone due to an increase in mRNA expression of corticotrophin-releasing hormone and activation of protein kinase C ζ/λ and δ in the placenta (see Supplemental Material, Table S3). A few *in vitro* studies have indicated that BPA affects placental cell proliferation. After 24 hr or 48 hr of culture, BPA (1–10 μM) increased apoptosis and decreased proliferation of cultured human trophoblast cells from first trimester placentas, whereas 10-μM BPA decreased cell viability (Morice at al. 2011). Similarly, after 24 hr, BPA (0.0877–8.77 μM) increased apoptosis in cultured human cytotrophoblasts (Benachour and Aris 2009). In addition, recent data from mouse studies suggest that BPA alters gene expression in the placenta (Susiarjo et al. 2013). Taken together, these *in vitro* data suggest that BPA may affect placental function, but additional animal and human studies are required to substantiate these data.

## Pregnancy Outcomes

Only one study has examined the association between BPA exposure and pregnancy outcome in humans. Specifically, in a nested case–control study of 60 pregnant women, Cantonwine et al. (2010) found a positive association between urinary BPA concentration and preterm birth (see Supplemental Material, Table S4). Given the limited information available, more studies are needed before making conclusions about whether BPA exposure is associated with adverse pregnancy outcomes in humans.

In contrast, several experimental studies have investigated the effects of BPA exposure on pregnancy outcomes in animal models, such as mice and rats, which are altrical species in which pups are born at a stage of development equivalent to midgestation in humans. As shown in Supplemental Material, Table S5, several studies have reported that low-dose BPA did not alter gestation length in gestationally plus neonatally exposed mice (Cabaton et al. 2011; Kobayashi et al. 2010), adult-exposed mice (Tyl et al. 2008), or gestationally plus neonatally exposed SD rats (Kobayashi et al. 2012). Cabaton et al. (2011) reported that low-dose BPA decreased the number of pregnancies and successful deliveries in gestationally exposed mice. BPA also decreased the percent of hatchings of chickens exposed *in ovo* to BPA (134 ng/kg) on day 4 on incubation (Yigit and Daglioglu 2010). Conversely, neither low- nor high-dose BPA affected the number of litters born to unexposed female SD rats mated to gestationally exposed males (Thuillier et al. 2009), suggesting that maternal, but not paternal, BPA exposure may influence successful delivery of offspring.

Many experimental studies have reported that low-dose BPA does not affect the number of live pups (Howdeshell et al. 2008; Kobayashi et al. 2010, 2012; Thuillier et al. 2009; Xi et al. 2011) or total number of delivered pups (Kobayashi et al. 2010, 2012; Nanjappa et al. 2012; Ryan et al. 2010; Tyl et al. 2008; Xi et al. 2011) in mice and rats. In a few studies, however, low-dose BPA exposure was reported to decrease the number of live pups born to prenatally plus neonatally exposed CD-1 mice (Cabaton et al. 2011) and Holtzman rats (Salian et al. 2009b). Low-dose BPA also decreased the total number of pups born to gestationally and neonatally exposed CD-1 mice (Cabaton et al. 2011). Interestingly, BPA acted differently from the positive control (diethylstilbestrol; DES), suggesting that the effects of BPA may differ from those of DES in CD-1 mice. Low-dose BPA also decreased the total number of pups born to unexposed female Holtzman rats mated to neonatally and gestationally exposed males of the same strain (Salian et al. 2009a, 2009b). In these two studies, BPA exposure decreased pup numbers after multiple litters, similar to DES. Collectively, these studies suggest that BPA exposure affects pregnancy outcomes in many, but not all, studies depending on experimental protocol. Clearly, more studies are required to determine why results differ and to determine whether BPA exposure does indeed affect pregnancy outcomes in animal models.

## Birth Weight

Human studies of BPA and birth weight are equivocal (see Supplemental Material, Table S4). In a cross-sectional study of 97 pregnant women, Chou et al. (2011) observed that women with serum BPA concentrations > 2.51 ng/mL had a higher risk for having male babies that were small for gestational age than did women with lower serum BPA concentrations. However, these authors found no associations with female babies (see Supplemental Material, Table S4). These results are consistent with a retrospective cohort study that included 587 births in which maternal and paternal occupational exposure to BPA were both associated with low birth weight and small gestational size; the association was stronger for maternal exposure than paternal exposure (Miao et al. 2011b). Further, in a Dutch population-based prospective cohort study of 219 pregnant women, urinary BPA was positively associated with lower growth rates and smaller head circumference (Snijder et al. 2013). Similarly, in a birth cohort study of 757 Korean pregnant women participating in the Mothers and Children’s Environmental Health (MOCEH) study, BPA was associated with increased birth weight in males and neonatal length in females (Lee BE et al. 2014). However, in a case–control study of 191 pregnant women, BPA had an inverse U-shaped association with birth weight (Philippat et al. 2012). In a cross-sectional study of 40 pregnant women, urinary BPA levels at delivery were not associated with birth weights of the offspring (Padmanabhan et al. 2008). Further, in a prospective cohort study of 404 mother–infant pairs, BPA exposure was not associated with increased birth weight (Wolff et al. 2008b). Given some of these discrepant results, future studies should assess whether BPA exposure is associated with birth weight.

In previous work, gestational exposure to low doses of BPA did not alter the birth weight of mice or rats. However, recent animal studies suggest that the effect of BPA on offspring birth weight is dose dependent (see Supplemental Material, Table S5). Tyl et al. (2008) reported that high-dose BPA increased the weight of offspring born to gestationally exposed mice. Conversely, low-dose BPA decreased the weight of offspring born to neonatally exposed mice (Nah et al. 2011). However, at doses below the U.S. EPA reference dose (50 μg/kg) (U.S. EPA 1993), BPA did not affect the weight of offspring born to gestationally plus neonatally exposed mice (Kobayashi et al. 2010) or rats (Howdeshell et al. 2008; Kobayashi et al. 2012; Nanjappa et al. 2012; Ryan et al. 2010), suggesting that BPA has a specific dose effect on birth weight of offspring.

## Sperm Production and Quality

Human studies of the effect of adult exposure to BPA on sperm quality are very limited (see Supplemental Material, Table S6). Studies of occupationally exposed men (Li et al. 2011) and men recruited from an infertility clinic (Meeker et al. 2010a) reported that higher urinary BPA levels were associated with a decrease in sperm count and motility. However, in a study of fertile men, urinary BPA concentration was not associated with changes in any semen parameters, but urinary BPA levels were significantly associated with markers of free testosterone (Mendiola et al. 2010). Although the results of these few epidemiological studies are consistent, there is insufficient evidence to draw conclusions about the association between BPA and semen quality in humans.

The earliest experimental studies of the effects of BPA on sperm suggested adverse effects on spermatogenesis in the adult following either prenatal or early postnatal exposure (reviewed by Richter et al. 2007). Subsequent experimental studies have provided further evidence that prenatal and early postnatal exposure to BPA adversely affect spermatogenesis and have provided new evidence that exposure in adult rodents affects sperm quality (see Supplemental Material, Table S7). In rodents, exposure during the time of testis development—either during gestation or in the early postnatal period—or to young adult males has been associated with a range of adverse effects in the adult testis. However, variability in exposure timing, species and strains, end points, and life stage makes a simple analysis of the data difficult. Despite the variation in study design, several findings have emerged repeatedly, including decreased sperm counts, increased apoptotic cells within the seminiferous tubules, and decreased sperm motility; alterations in hormone levels and/or steroidogenic enzymes; and evidence of sperm DNA damage.

The evidence that BPA exposure adversely affects sperm production and quality in the adult rodent is supported by data from recent studies (see Supplemental Material, Table S7). Gestational exposure to low-dose BPA resulted in a decreased number of elongated spermatids present in seminiferous tubules in pubertal ICR mice (Okada and Kai 2008) and decreased sperm counts in Holtzman rats (Salian et al. 2009a). Similarly, both low- and high-dose BPA exposure during early postnatal development or around the time of puberty increased apoptosis and/or decreased spermatogenesis in male mice and rats (Li et al. 2009; Liu et al. 2013; Qiu et al. 2013; Wang et al. 2010). Further, adult rats exposed to low-dose BPA for either 6 or 14 days showed increased apoptosis and decreased sperm counts (Jin et al. 2013; Tiwari and Vanage 2013).

In addition, a number of studies using various routes of exposure (oral and subcutaneous) and different exposure times (embryonic, fetal, perinatal, and adult) have reported that low-dose BPA impairs sperm motility in rats and mice (Dobrzynska and Radzikowska 2013; Minamiyama et al. 2010; Salian et al. 2009a; Tainaka et al. 2012; Tiwari and Vanage 2013) (see Supplemental Material, Table S7). Interestingly, Minamiyama et al. (2010) reported that the BPA-induced decrease in rat sperm motility was prevented by coadministration of the antioxidant *n*-acetyl cysteine, suggesting that impaired sperm motility may be related to increases in reactive oxygen species (ROS). As reviewed by Ross et al. (2010), antioxidant use has been suggested to increase sperm quality in men and increase pregnancy rates of infertile couples.

Spermatogenesis is dependent upon androgens, and changes that impact the endocrine environment of the testes have the potential to adversely affect sperm production and quality. Endocrine-related changes have been reported in several experimental studies: Rodent studies of gestational exposure alone and continuous gestational–neonatal exposure to BPA have reported evidence of impaired steroidogenesis, a decreased number of steroid receptors, or a decrease in *Star* expression (see Supplemental Material, Table S7). However, the most compelling data have come from studies of male rats exposed in the postnatal/adult period: Most of these studies reported a decrease in testosterone levels and/or steroidogenic enzymes in BPA-exposed males (low or high dose) compared with controls (Castro et al. 2013; D’Cruz et al. 2012a; El-Beshbishy et al. 2012; Jin et al. 2013; Nakamura et al. 2010; Wu et al. 2011). Only one study in ICR mice reported no change in testosterone levels in low-dose-BPA–exposed males compared with controls, despite the fact that the exposed males had impaired spermatogenesis (Okada and Kai 2008).

In addition to endocrine changes, sperm DNA damage has been reported in BPA-exposed rodents (see Supplemental Material, Table S7). Consistent with studies before 2007 (Chitra et al. 2003; Kabuto et al. 2003, 2004), recent studies have shown that continuous exposure to low-dose BPA exposure induces DNA breaks and the production of ROS in mice and rats (Anjum et al. 2011; Dobrzynska and Radzikowska 2013; Fang et al. 2013; Liu et al. 2013; Minamiyama et al. 2010; Rashid et al. 2009; Wu et al. 2013) (see Supplemental Material, Table S7). Tiwari and Vanage (2013) reported that oral exposure to low-dose BPA for 6 days also induced sperm DNA damage. Similarly, oral low-dose BPA exposure has been reported to decrease testicular glucose levels and the expression and translation of glucose transporter-8 in spermatocytes and spermatids and to increase testicular hydrogen peroxide levels in Wistar rats (D’Cruz et al. 2012a, 2012b) and oxidative stress in SD rats (Qiu et al. 2013).

Unfortunately, the relevance of the doses used in the experimental studies to human exposure is not certain because internal or serum BPA levels were not determined in the studies. Nevertheless, these data add to the evidence that BPA exposure in the adult rodent adversely impacts the testis and sperm quality at levels below the LOAEL of 50 mg/kg. Although similar reproductive effects have been reported in multiple studies, a few studies failed to find any adverse reproductive effects in rodent models after gestational plus neonatal BPA exposure (Howdeshell et al. 2008; Kobayashi et al. 2010; LaRocca et al. 2011; Tyl et al. 2008) (see Supplemental Material, Table S7). These discordant findings may be related to a variety of factors, including dose, exposure route, internal dose, timing, and selected end points. Nevertheless, commonalities among studies finding no adverse effects raise several concerns. First, two of the four studies used the same inbred mouse strain, C57BL/6 (LaRocca et al. 2011; Kobayashi et al. 2010), suggesting that this strain may be insensitive or less sensitive to the effects of BPA on sperm than other strains. Second, dietary exposure has been used in several no-effect studies, but it was not used in any studies reporting adverse effects. These results add to the concern about the role that route of exposure plays in BPA studies and substantiates calls to replace exposure route with measurements of internal dose. Indeed, recent studies have shown that the route of BPA administration results in markedly different ratios of unconjugated (bioactive) to conjugated BPA and provided evidence that much of human exposure must be from non-oral routes (Gayrard et al. 2013; vom Saal et al. 2014). Last, two of the studies (Howdeshell et al. 2008; Tyl et al. 2008) focused on end points such as body/organ weight, which alone may not accurately measure effects on male fertility. Indeed, some studies reporting detrimental effects (e.g., increased germ cell apoptosis, reduction in sperm counts) did not find significant changes in testis or epididymal weight (see Supplemental Material, Table S7). Nevertheless, these negative findings underscore the need for comparative experimental studies involving different strains of animals and routes of exposure. In addition, assessing the importance of effects during different life stages is important, particularly because Newbold et al. (2009) identified abnormalities in middle age that were not seen in young adult female rodents.

## Steroidogenesis in Males

Three epidemiological studies investigated the association between BPA exposure and serum hormone levels in men (see Supplemental Material, Table S6). In a study of 307 Italian men participating in the INChianti study (a prospective, population-based study of adults living in Chianti, Italy), Galloway et al. (2010) observed an association between increased urinary BPA concentrations and increased serum testosterone levels, but not estradiol levels. This observation was not supported by two cross-sectional studies of 167 and 302 fertile men, which reported no correlation between BPA exposure and testosterone levels (Meeker et al. 2010b; Mendiola et al. 2010). However, Mendiola et al. (2010) found associations between urinary BPA concentrations and decreased free androgen index (FAI), the ratio of FAI to luteinizing hormone, and the ratio of free testosterone to luteinizing hormone in male partners of pregnant women. Meeker et al. (2010b) found an association between high BPA concentrations and increased serum levels of follicle-stimulating hormone, and between high BPA concentrations and decreased levels of inhibin B, the ratio of follicle-stimulating hormone to inhibin B, and the ratio of estradiol to testosterone. These data suggest that BPA can alter steroid hormone pathways in men; however, because of limited association studies and likely multifactorial responses produced by BPA, more studies are needed to determine how BPA affects steroidogenesis. Further, because most studies conducted before 2007 focused on infertile men, it is important to conduct studies on fertile men to confidently determine if BPA affects male steroidogenesis and whether findings in infertile men can be extrapolated to the general population.

Experimental studies indicate that BPA exposure decreases hormone levels in male animals, but the data are discordant (see Supplemental Material, Table S7). Low-dose BPA was reported to decrease testosterone levels in gestationally through postnatally exposed CD-1 mice (Xi et al. 2011) but not in adult C57BL/6 mice exposed *in utero* (LaRocca et al. 2011). Low-dose BPA decreased testosterone levels in gestationally or neonatally exposed Holtzman rats (Salian et al. 2009a, 2009b), adult-exposed albino rats (El Beshbishy et al. 2012), and adult-exposed Wistar rats (D’Cruz et al. 2012a). Conversely, gestational plus neonatal exposure to low-dose BPA did not affect testosterone levels in Long Evans (LE) rats (Howdeshell et al. 2008) or SD rats (Kobayashi et al. 2012; Qiu et al. 2013). However, although Sánchez et al. (2013) reported that low dose BPA did not decrease testosterone in Wistar rats, low-dose BPA decreased dihydrotestosterone levels (Sánchez et al. 2013). Mechanistic underpinnings for the ability of BPA to inhibit testosterone levels may be found in the fact that BPA decreases expression of steroidogenic enzymes (Horstman et al. 2012; Nakamura et al. 2010; Qiu et al. 2013; Xi et al. 2011) and levels of follicle-stimulating hormone, which are required to directly and indirectly stimulate steroidogenesis (Salian et al. 2009a, 2009b). Collectively, these data indicate that BPA exposure decreases sex steroid hormone levels in male rodents, but strain and species, as well as other confounders (e.g., time, route of exposure, age at analysis), may modulate the sensitivity to BPA effects.

Because Leydig cells are responsible for most steroid hormone production in males, experimental studies have been designed to examine whether BPA directly affects Leydig cells. In pubertal Wistar/ST rats, continuous exposure to high-dose BPA was reported to decrease cell numbers and expression of steroidogenic enzymes in the Leydig cells. (Nakamura et al. 2010). Conversely, low-dose BPA increased Leydig cell numbers in gestationally plus neonatally exposed LE rats in adulthood by up-regulating mitogenic factors (Nanjappa et al. 2012). Although BPA exposure increased Leydig cell proliferation, it did not alter circulating testosterone levels. In fact, in *in vitro* Leydig cell isolates from adult-exposed males, low-dose BPA decreased the expression of steroidogenic enzymes and testosterone production (Nanjappa et al. 2012). Similarly, BPA decreased testosterone levels in human, mouse, and rat fetal testes *in vitro* (N’Tumba-Byn et al. 2013). In another study, Thuillier et al. (2009) reported that gestational exposure to low- and high-dose BPA increased Leydig cell numbers without affecting serum testosterone levels in SD rats in adulthood. Collectively, these data strongly suggest that BPA negatively affects gonadal function through changes in steroid-synthesizing cells/enzymes, which then affect steroid synthesis and circulating steroid levels.

## Anogenital Distance and Cryptorchidism

Disruptions in testicular testosterone production during development may lead to phenotypic abnormalities after birth, such as shortened anogenital distance (AGD) and undescended testes. In a retrospective occupational study of 587 Chinese children, Miao (2011b) found a dose-dependent association between parental occupational exposure to BPA and shortened AGD in male offspring (see Supplemental Material, Table S4). This inverse correlation was strengthened with increasing maternal BPA concentrations (Miao et al. 2011a). Interestingly, Fenichel et al. (2012) observed that BPA concentrations in cord blood samples were not correlated with cryptorchidism in a matched case–control study of 152 newborn males. Although these studies suggest that BPA exposure may be associated with shortened AGD and undescended testes, it is important to confirm these findings in other epidemiological studies.

In several animal studies, BPA exposure did not affect AGD, but it did influence testes descent (see Supplemental Material, Table S7). In four recent studies, gestational or gestational plus neonatal exposure to low-dose BPA did not alter AGD in male rodents (Howdeshell et al. 2008; Kobayashi et al. 2010, 2012; LaRocca et al. 2011). In another study, Tyl et al. (2008) reported that gestational exposure to high-dose BPA decreased absolute AGD in F_1_ CD-1 males at birth, but not in the F_2_ generation and not when normalized to relative body size. In the same study, high-dose BPA delayed preputial separation and increased the incidence of treatment-related undescended testes in F_1_ and F_2_ offspring. Notably, the doses used by Tyl et al. (2008) were very high and not physiologically relevant to human exposure. Collectively, these data suggest that BPA exposure may not affect AGD in animal models, but it may affect other developmental aspects such as undescended testes.

## Male Urinary Tract

To date, no epidemiological studies have reported associations between BPA exposure and alterations in the male urinary tract, and only a few experimental studies have evaluated the effects of BPA on the male urinary tract (see Supplemental Material, Table S7). In the adult rat prostate, low- and high-dose BPA exposure has been reported to adversely increase clusterin levels and expression of aromatase, while decreasing levels of 5α-reductase type 1 and 2 (Castro et al. 2013; De Flora et al. 2011; Sánchez et al. 2013). Interestingly, Castro et al. (2013) and De Flora et al. (2011) reported that BPA exposure increased the plasma estradiol to testosterone ratio, which has been implicated in the development of benign prostate hyperplasia (Nicholson et al. 2012; Nicholson and Ricke 2011). Further, gestational exposure to low-dose BPA increased gene expression of androgen receptor, *Esr1*, aromatase, and estrogen-related receptor γ in the mouse prostate (Arase et al. 2011).

Other studies have indicated that BPA exposure plays a role in prostate pathogenesis in animal models. Prins et al. (2011) reported that neonatal exposure of rats to low-dose BPA increased the incidence of the precursor for prostate cancer, prostate intraepithelial neoplasia, in adulthood. Further, Wu et al. (2011) reported that in rats with induced prostatic hyperplasia, high-dose BPA increased prostate gland mass and increased relative weight of the dorsolateral prostate lobe. High-dose BPA exposure also increased epithelial cell heights in the ventral prostate and dorsolateral prostate lobes (Wu et al. 2011). Last, neonatal exposure to low-dose BPA led to transient and permanent hypomethylations in the rat epigenome, implicated in the manifestation of prostatic carcinogenesis (Tang et al. 2012). Collectively, these studies support the concept that both low and high doses of BPA promote changes in steroidogenic pathways and morphology of the prostate, which may in turn affect homeostasis and pathogenesis.

Currently, no information is available about whether BPA exposure causes benign urologic disease. However, studies have shown that estrogens and estrogen receptor pathways negatively affect the lower urinary tract (Nicholson et al. 2012; Ricke et al. 2008; Wang et al. 2007; Willingham and Baskin 2007), supporting a potential role for estrogen-like molecules, including BPA, in the manifestation of urological diseases. Future experimental studies evaluating the effects of BPA on the lower urinary tract, as well as epidemiological studies in humans, need to gain insight into the role BPA plays in disease processes in the lower male urinary tract.

## Puberty and Sexual Receptivity

Two epidemiological studies investigated the effect of BPA on puberty and measured BPA levels in girls at similar ages (see Supplemental Material, Table S2). In these studies, one of 1,151 girls 6–8 years of age (Wolff et al. 2010) and another of 192 girls 9 years of age (Wolff et al. 2008a), BPA exposure was not associated with accelerated breast or pubic hair development. In a study of 82 patients with precocious puberty and 32 patients without precocious puberty, BPA exposure was not associated with precocious puberty (Lee SH et al. 2014). However, in a study of 110 girls with precocious puberty and 100 girls without precocious puberty, BPA was associated with increased uterine and ovarian volume (Qiao et al. 2010). These studies suggest that BPA exposure may not be associated with onset of puberty in girls, but given the limited number of studies, these results should be confirmed in future studies.

In animal models, the effects of BPA on factors such as puberty onset and sexual receptivity are equivocal (see Supplemental Material, Table S8). In one study (Ryan et al. 2010), low-dose BPA did not affect the timing of puberty onset as measured by vaginal opening in LE rats orally exposed to BPA gestationally plus neonatally. Conversely, low- and high-dose BPA exposure accelerated vaginal opening in neonatally exposed ICR mice (Nah et al. 2011), SD rats (Fernández et al. 2009), and LE rats (Adewale et al. 2009). Low-dose BPA also decreased the time neonatally exposed ICR mice and SD rats spent in estrus (Fernández et al. 2009; Nah et al. 2011). However, low-dose BPA did not affect estrous cyclicity in gestationally plus neonatally exposed CD-1 mice and LE rats (Adewale et al. 2009; Tyl et al. 2008). Further, low-dose BPA had no effect on lordosis behavior in gestationally plus neonatally exposed LE rats (Ryan et al. 2010) or neonatally exposed female LE rats (Adewale et al. 2009). Collectively, these studies indicate that the effects of BPA on the onset of puberty and sexual receptivity in animal models are unclear and likely differ depending on strain and species. Further studies should examine differences in puberty onset and sexual receptivity in various strains and species of animal models.

## Sexual Dysfunction

Few epidemiological or experimental studies have examined the association between BPA exposure and sexual dysfunction. One cross-sectional study of 425 men occupationally exposed to BPA reported an increased risk of self-reported impaired sexual abilities compared to 284 unexposed men (Li et al. 2010) (see Supplemental Material, Table S6). Some animal studies have indicated that BPA may impair sexual ability (see Supplemental Material, Table S8). Low-dose BPA increased the time taken for copulation in neonatally or gestationally exposed Holtzman rats (Salian et al. 2009a, 2009b) and increased latency to insemination in perinatally exposed CF-1 mice (deCatanzaro et al. 2013). Low-dose BPA also decreased intromission and ejaculations of perinatally exposed male CF-1 mice mated to unexposed females (deCatanzaro et al. 2013). However, in another study, low- or high-dose BPA did not affect the time taken for copulation in gestationally plus neonatally exposed adult CD-1 mice or their offspring (Tyl et al. 2008). Given the limited number of studies conducted on BPA and sexual function and the equivocal nature of the results, it is not possible to make firm conclusions on the effects of BPA on sexual function. However, it is important to note that all but one study conducted to date suggest that BPA negatively impacts sexual function.

## Conclusions

Here we summarized the strength of the evidence for associations between BPA and adverse reproductive outcomes based on literature published from 2007 through 2013. The data presented in this review build on the overall conclusions of the expert panel report (vom Saal et al. 2007) that the widespread effects of BPA in experimental animal studies are a concern for overall human health and may be involved in human reproductive disease. Similar to the 2007 expert panel report, we considered the evidence to be strong when multiple studies in multiple species indicated a similar effect of BPA on a reproductive tissue or end point, even if concordance was not 100% across all studies, given that species and strain differences can lead to differences in dose response and magnitude of effect. These conclusions, however, are not to be considered definitive without further investigation, especially with the gaps in clear results detailed throughout the review. In the experimental studies, strong, definitive conclusions often were difficult because study designs were so different. Experimental studies rarely used the same doses, timing, positive controls, and exposure routes; thus, the effects of BPA on exposure among animal strains and species could not effectively be compared. However, one unifying strength of these studies related to human health is that most of the studies we evaluated used BPA doses below the LOAEL. In the epidemiological studies, strong conclusions were difficult to determine because of study design and exposure parameters. For example, exposure assessments in most human studies relied on a single urine sample, which may introduce exposure misclassification and attenuate associations if they are present. Given the continuous and variable exposure to BPA, a single urine sample may not represent longer-term exposure or exposure in the relevant etiological window. Finally, most of the human studies were cross-sectional, making it difficult to discern the temporal relationship of exposure with response. These limitations affect the interpretation of human studies. Future human studies need to consider improvements in exposure assessment to represent longer-term BPA exposure assessed at etiologically relevant windows. Given recent data, it will be critical in future studies to assess effects of different routes of exposure and to analyze effects at different stages in the life of the exposed individual. In addition, for experimental studies, the inclusion of positive controls should be considered essential.

However, given the data included in this review, we have drawn some insights and conclusions that add to the conclusions drawn based on reviews of the BPA literature prior to 2007, categorized by the strength of evidence presented.

1. Strong evidence exists that
  BPA is an ovarian toxicant in animal models and women. It adversely affects the onset of meiosis in ovaries from both animal models and humans, interferes with germ cell nest breakdown in animal models, accelerates follicle transition in several animal species, alters steroidogenesis in multiple animal models and in women, and reduces oocyte quality in animal models and in women undergoing IVF.
  BPA is a uterine toxicant in animal models because it impairs uterine endometrial cellular proliferation, decreases uterine receptivity, alters gene expression, and increases implantation failure in several strains and species. However, human studies have not adequately addressed these end points.
  BPA is a prostate toxicant in animal models, impairing the steroidogenic capacity and altering dorsal and ventral lobe morphology, potentially leading to prostate pathogenesis. Human studies, however, are lacking.
  The effects of BPA on the reproductive system are variable and evident at doses below the LOAEL of 50 mg/kg and the proposed safe level of 50 μg/kg/day.
2. Limited evidence exists that
  Relative to the impact of BPA on birth rate, birth weight, and length of gestation, human data on birth weight are inconsistent; although animal studies suggest such an association, there are limited human studies on gestational length and preterm birth.
  BPA exposure is associated with hyperandrogenism, such as in PCOS in women. However, data in rodent models do not support the development of symptoms of human PCOS.
  BPA is a testicular toxicant in animal models because it decreases sperm quality and motility, causes oxidative stress, and alters steroidogenesis. Human data, however, are inconsistent.
  BPA is associated with impaired implantation in women undergoing IVF. The associations between BPA and implantation failure in women may be due to the effects of BPA on the embryo or the uterus, or both.
  BPA exposure in male rats is associated with implantation failure in nonexposed female rats. Human data, however, are lacking.
  BPA is associated with sexual dysfunction among men exposed to high occupational levels and in experimental studies on males.
3. There is insufficient evidence to draw conclusions regarding effects of BPA on the oviduct, placenta, and pubertal development.
4. Future studies need to
  Consider the critical period of differentiation of the organ system in question and the reproductive life span of the animal model or human.
  Use continuous exposure to BPA in view of the ubiquitous and continuous exposure to BPA in humans.
  Target internal dose levels of BPA that are achieved by human exposures.
  Recognize the potential interaction of BPA with other hormone-altering chemicals and lifestyle factors such as diet and stress.
  Distinguish organizational (permanent) versus activational (transient) effects of BPA.
  Determine whether preterm and maternal morbidities (e.g., preeclampsia, gestational diabetes), as well as paternal factors, modify or mediate the effects of BPA on the reproductive system.

## Supplemental Material

(770 KB) PDF

## References

[r1] Adewale HB, Jefferson WN, Newbold RR, Patisaul HB (2009). Neonatal bisphenol-A exposure alters rat reproductive development and ovarian morphology without impairing activation of gonadotropin-releasing hormone neurons.. Biol Reprod.

[r2] Aghajanova L, Giudice LC (2011). Effect of bisphenol A on human endometrial stromal fibroblasts *in vivo*.. Reprod Biomed Online.

[r3] Aldad TS, Rahmani N, Leranth C, Taylor HS (2011). Bisphenol-A exposure alters endometrial progesterone receptor expression in the nonhuman primate.. Fertil Steril.

[r4] An BS, Ahn HJ, Kang HS, Jung EM, Yang H, Hong EJ (2013). Effects of estrogen and estrogenic compounds, 4-*tert*-octylphenol, and bisphenol A on the uterine contraction and contraction-associated proteins in rats.. Mol Cell Endocrinol.

[r5] Anjum S, Rahman S, Kaur M, Ahmad F, Rashid H, Ansari RA (2011). Melatonin ameliorates bisphenol A-induced biochemical toxicity in testicular mitochondria of mouse.. Food Chem Toxicol.

[r6] Arase S, Ishii K, Igarashi K, Aisaki K, Yoshio Y, Matsushima A (2011). Endocrine disrupter bisphenol A increases in situ estrogen production in the mouse urogenital sinus.. Biol Reprod.

[r7] Benachour N, Aris A (2009). Toxic effects of low doses of bisphenol-A on human placental cells.. Toxicol Appl Pharmacol.

[r8] Berger RG, Foster WG, DeCatanzaro D (2010). Bisphenol-A exposure during the period of blastocyst implantation alters uterine morphology and perturbs measures of estrogen and progesterone receptor expression in mice.. Reprod Toxicol.

[r9] Berger RG, Shaw J, deCatanzaro D (2008). Impact of acute bisphenol-A exposure upon intrauterine implantation of fertilized ova and urinary levels of progesterone and 17β-estradiol.. Reprod Toxicol.

[r10] Bloom MS, Kim D, vom Saal FS, Taylor JA, Cheng G, Lamb JD (2011a). Bisphenol A exposure reduces the estradiol response to gonadotropin stimulation during *in vivo* fertilization.. Fertil Steril.

[r11] Bloom MS, vom Saal FS, Kim D, Taylor JA, Lamb JD, Fujimoto VY (2011b). Serum unconjugated bisphenol A concentrations in men may influence embryo quality indicators during *in vivo* fertilization.. Environ Toxicol Pharmacol.

[r12] Bosquiazzo VL, Varayoud J, Munoz-de-Toro M, Luque EH, Ramos JG (2010). Effects of neonatal exposure to bisphenol A on steroid regulation of vascular endothelial growth factor expression and endothelial cell proliferation in the adult rat uterus.. Biol Reprod.

[r13] Bredhult C, Sahlin L, Olovsson M (2009). Gene expression analysis of human endometrial endothelial cells exposed to bisphenol A.. Reprod Toxicol.

[r14] Brieño-Enriquez MA, Reig-Viader R, Cabero L, Toran N, Martinez F, Roig I (2012). Gene expression is altered after bisphenol A exposure in human fetal oocytes *in vitro*.. Mol Hum Reprod.

[r15] Brieño-Enriquez MA, Robles P, Camats-Tarruella N, Garcia-Cruz R, Roig I, Cabero L (2011). Human meiotic progression and recombination are affected by bisphenol A exposure during *in vitro* human oocyte development.. Hum Reprod.

[r16] Bromer JG, Zhou Y, Taylor MB, Doherty L, Taylor HS (2010). Bisphenol-A exposure *in utero* leads to epigenetic alterations in the developmental programming of uterine estrogen response.. FASEB J.

[r17] Buck Louis GM, Peterson CM, Chen Z, Croughan M, Sundaram R, Stanford J (2013). Bisphenol A and phthalates and endometriosis: the Endometriosis: Natural History, Diagnosis and Outcomes Study.. Fertil Steril.

[r18] Cabaton NJ Wadia PR Rubin BS Zalko D Schaeberle CM Askenase MH 2011 Perinatal exposure to environmentally relevant levels of bisphenol A decreases fertility and fecundity in CD-1 mice. Environ Health Perspect 119 547 552; 10.1289/ehp.1002559 21126938 PMC3080939

[r19] Cantonwine D Meeker JD Hu H Sánchez BN Lamadrid-Figueroa H Mercado-García A 2010 Bisphenol A exposure in Mexico City and risk of prematurity: a pilot nested case control study. Environ Health 9 62; 10.1186/1476-069X-9-62 20955576 PMC2965706

[r20] Castro B Sánchez P Torres JM Preda O del Moral RG Ortega E 2013 Bisphenol A exposure during adulthood alters expression of aromatase and 5α-reductase isozymes in rat prostate. PLoS One 8 2 e55905; 10.1371/journal.pone.0055905 23405234 PMC3566099

[r21] Chao HH, Zhang XF, Chen B, Pan B, Zhang LJ, Li L (2012). Bisphenol A exposure modifies methylation of imprinted genes in mouse oocytes via the estrogen receptor signaling pathway.. Histochem Cell Biol.

[r22] Chitra KC, Latchoumycandane C, Mathur PP (2003). Induction of oxidative stress by bisphenol A in the epididymal sperm of rats.. Toxicology.

[r23] Chou WC Chen JL Lin CF Chen YC Shih FC Chuang CY 2011 Biomonitoring of bisphenol A concentrations in maternal and umbilical cord blood in regard to birth outcomes and adipokine expression: a birth cohort study in Taiwan. Environ Health 10 94; 10.1186/1476-069X-10-94 22050967 PMC3225308

[r24] Cobellis L, Colacurci N, Trabucco E, Carpentiero C, Grumetto L (2009). Measurement of bisphenol A and bisphenol B levels in human blood sera from healthy and endometriotic women.. Biomed Chromatogr.

[r25] D’Cruz SC, Jubendradass R, Jayakanthan M, Rani SJ, Mathur PP (2012a). Bisphenol A impairs insulin signaling and glucose homeostasis and decreases steroidogenesis in rat testis: an *in vivo* and *in silico study*.. Food Chem Toxicol.

[r26] D’Cruz SC, Jubendradass R, Mathur PP (2012b). Bisphenol A induces oxidative stress and decreases levels of insulin receptor substrate 2 and glucose transporter 8 in rat testis.. Reprod Sci.

[r27] deCatanzaro D, Berger RG, Guzzo AC, Thorpe JB, Khan A (2013). Perturbation of male sexual behavior in mice (*Mus musculus*) within a discrete range of perinatal bisphenol-A doses in the context of a high- or low-phytoestrogen diet.. Food Chem Toxicol.

[r28] De Flora S, Micale RT, La Maestra S, Izzotti A, D’Agostini F, Camoirano A (2011). Upregulation of clusterin in prostate and DNA damage in spermatozoa from bisphenol A-treated rats and formation of DNA adducts in cultured human prostatic cells.. Toxicol Sci.

[r29] Dobrzynska MM, Radzikowska J (2013). Genotoxicity and reproductive toxicity of bisphenol A and X-ray/bisphenol A combination in male mice.. Drug Chem Toxicol.

[r30] Ehrlich S, Calafat AM, Humblet O, Smith T, Hauser R (2014). Handling of thermal receipts as a source of exposure to bisphenol A.. JAMA.

[r31] Ehrlich S, Williams PL, Hauser R, Missmer SA, Peretz J, Calafat AM (2013). Urinary bisphenol A concentrations and cytochrome P450 19 A1 (*Cyp19*) gene expression in ovarian granulosa cells: an *in vivo* human study.. Reprod Toxicol.

[r32] Ehrlich S Williams PL Missmer SA Flaws JA Berry KF Calafat AM 2012a Urinary bisphenol A concentrations and implantation failure among women undergoing *in vitro* fertilization. Environ Health Perspect 120 978 983; 10.1289/ehp.1104307 22484414 PMC3404656

[r33] Ehrlich S, Williams PL, Missmer SA, Flaws JA, Ye X, Calafat AM (2012b). Urinary bisphenol A concentrations and early reproductive health outcomes among women undergoing IVF.. Hum Reprod.

[r34] Eichenlaub-Ritter U, Vogt E, Cukurcam S, Sun F, Pacchierotti F, Parry J (2008). Exposure of mouse oocytes to bisphenol A causes meiotic arrest but not aneuploidy.. Mutat Res.

[r35] El Beshbishy HA Aly HA El Shafey M 2012 Lipoic acid mitigates bisphenol A-induced testicular mitochondrial toxicity in rats. Toxicol Ind Health; 10.1177/0748233712446728 22623521

[r36] Fang Y Zhou Y Zhong Y Gao X Tan T 2013 Effect of vitamin E on reproductive functions and anti-oxidant activity of adolescent male mice exposed to bisphenol A [in Chinese] Wei Sheng Yan Jiu 42 18 22 23596702

[r37] Fenichel P, Dechaux H, Harthe C, Gal J, Ferrari P, Pacini P (2012). Unconjugated bisphenol A cord blood levels in boys with descended or undescended testes.. Hum Reprod.

[r38] Fernández M Bianchi M Lux-Lantos V Libertun C 2009 Neonatal exposure to bisphenol A alters reproductive parameters and gonadotropin releasing hormone signaling in female rats. Environ Health Perspect 117 757 762; 10.1289/ehp.0800267 19479018 PMC2685838

[r39] Fernández M Bourguignon N Lux-Lantos V Libertun C 2010 Neonatal exposure to bisphenol A and reproductive and endocrine alterations resembling the polycystic ovarian syndrome in adult rats. Environ Health Perspect 118 1217 1222; 10.1289/ehp.0901257 20413367 PMC2944080

[r40] Fujimoto VY, Kim D, vom Saal FS, Lamb JD, Taylor JA, Bloom MS (2011). Serum unconjugated bisphenol A concentrations in women may adversely influence oocyte quality during *in vivo* fertilization.. Fertil Steril.

[r41] Galloway T Cipelli R Guralnik J Ferrucci L Bandinelli S Corsi AM 2010 Daily bisphenol A excretion and associations with sex hormone concentrations: results from the InCHIANTI adult population study. Environ Health Perspect 118 1603 1608; 10.1289/ehp.1002367 20797929 PMC2974700

[r42] Gayrard V Lacroix MZ Collet SH Viguié C Bousquet-Melou A Toutain PL 2013 High bioavailability of bisphenol A from sublingual exposure. Environ Health Perpect 121 951 956; 10.1289/ehp.1206339 PMC3734497 23761051

[r43] Grasselli F, Baratta L, Baioni L, Bussolati S, Ramoni R, Grolli S (2010). Bisphenol A disrupts granulosa cell function.. Domest Anim Endocrinol.

[r44] Hampton T (2013). NIH panel: name change, new priorities advised for polycystic ovary syndrome.. JAMA.

[r45] Hiyama M, Choi EK, Wakitani S, Tachibana T, Khan H, Kusakabe KT (2011). Bisphenol-A (BPA) affects reproductive formation across generations in mice.. J Vet Med Sci.

[r46] Horstman KA, Naciff JM, Overmann GJ, Foertsch LM, Richardson BD, Daston GP (2012). Effects of transplacental 17-α-ethynyl estradiol or bisphenol A on the developmental profile of steroidogenic acute regulatory protein in the rat testis.. Birth Defects Res B Dev Reprod Toxicol.

[r47] Howdeshell KL, Furr J, Lambright CR, Wilson VS, Ryan BC, Gray LE (2008). Gestational and lactational exposure to ethinyl estradiol, but not bisphenol A, decreases androgen-dependent reproductive organ weights and epididymal sperm abundance in the male Long Evans hooded rat.. Toxicol Sci.

[r48] Hunt PA, Koehler KE, Susiarjo M, Hodges CA, Illagan A, Voigt RC (2003). Bisphenol A exposures causes meiotic aneuploidy in the female mouse.. Curr Biol.

[r49] Hunt PA, Lawson C, Gieske M, Murdoch B, Smith H, Marre A (2012). Bisphenol A alters early oogenesis and follicle formation in the fetal ovary of the rhesus monkey.. Proc Natl Acad Sci USA.

[r50] Hunt PA, Susiarjo M, Rubio C, Hassold TJ (2009). The bisphenol A experience: a primer for the analysis of environmental effects on mammalian reproduction.. Biol Reprod.

[r51] Iguchi T, Fukazawa Y, Uesugi Y, Taksugi N (1990). Polyovular follicles in mouse ovaries exposed neonatally to diethylstilbestrol in vivo and in vitro.. Biol Reprod.

[r52] Jin P, Wang X, Chang F, Bai Y, Li Y, Zhou R (2013). Low dose bisphenol A impairs spermatogenesis by suppressing reproductive hormone production and promoting germ cell apoptosis in adult rats.. J Biomed Res.

[r53] Kabuto H, Amakawa M, Shishibori T (2004). Exposure to bisphenol A during embryonic/fetal life and infancy increases oxidative injury and cause underdevelopment of the brain and testes in mice.. Life Sci.

[r54] Kabuto H, Hasuike S, Minagawa N, Shishibori T (2003). Effects of bisphenol A on the metabolisms of active oxygen species in mouse tissues.. Environ Res.

[r55] Kandaraki E, Chatzigeorgiou A, Livadas S, Palioura E, Economou F, Koutsilieris M (2011). Endocrine disruptors and polycystic ovary syndrome (PCOS): elevated serum levels of bisphenol A in women with PCOS.. J Clin Endocrinol Metab.

[r56] Kobayashi K, Kubota H, Ohtani K, Hojo R, Miyagawa M (2012). Lack of effects for dietary exposure of bisphenol A during *in utero* and lactational periods on reproductive development in rat offspring.. J Toxicol Sci.

[r57] Kobayashi K, Ohtani K, Kubota H, Miyagawa M (2010). Dietary exposure to low doses of bisphenol A: effects on reproduction and development in two generations of C57BL/6J mice.. Congenit Anom (Kyoto).

[r58] LaRocca J, Boyajian A, Brown C, Smith SD, Hixon M (2011). Effects of in utero exposure to bisphenol A or diethylstilbestrol on the adult male reproductive system.. Birth Defects Res B Dev Reprod Toxicol.

[r59] Lawson C, Gieske M, Murdoch B, Ye P, Li Y, Hassold T (2011). Gene expression in the fetal mouse ovary is altered by exposure to low doses of bisphenol A.. Biol Reprod.

[r60] Lee BE Park H Hong YC Ha M Kim Y Chang N 2014 Prenatal bisphenol A and birth outcomes: MOCEH (Mothers and Children’s Environmental Health) study. Int J Hyg Environ Health 217 2–3 328 334; 10.1016/j.ijheh.2013.07.005 23911140

[r61] Lee SG Kim JY Chung JY Kim YJ Park JE Oh S 2013 Bisphenol A exposure during adulthood causes augmentation of follicular atresia and luteal regression by decreasing 17β-estradiol synthesis via downregulation of aromatase in rat ovary. Environ Health Perspect 121 663 669; 10.1289/ehp.1205823 23512349 PMC3672913

[r62] Lee SH Kang SM Choi MH Lee J Park MJ Kim SH Lee WY Hong J Chung BC 2014 Changes in steroid metabolism among girls with precocious puberty may not be associated with urinary levels of bisphenol A. Reprod Toxicol 44 1 6; 10.1016/j.reprotox.2013.03.008 23557689

[r63] Lenie S, Cortvrindt R, Eichenlaub-Ritter U, Smitz J (2008). Continuous exposure to bisphenol A during in vivo follicular development induces meiotic abnormalities.. Mutat Res.

[r64] Li DK, Zhou Z, Miao M, He Y, Wang J, Ferber J (2011). Urine bisphenol-A (BPA) level in relation to semen quality.. Fertil Steril.

[r65] Li DK, Zhou Z, Qing D, He Y, Wu T, Miao M (2010). Occupational exposure to bisphenol-A (BPA) and the risk of self-reported male sexual dysfunction.. Hum Reprod.

[r66] Li Y Zhang W Liu J Wang W Li H Zhu J 2014 Prepubertal bisphenol A exposure interferes with ovarian follicle development and its relevant gene expression. Reprod Toxicol 44 33 40; 10.1016/j.reprotox.2013.09.002 24051130

[r67] Li YJ, Song TB, Cai YY, Zhou JS, Song X, Zhao X (2009). Bisphenol A exposure induces apoptosis and upregulation of Fas/FasL and caspase-3 expression in the testes of mice.. Toxicol Sci.

[r68] Liu C Duan W Li R Xu S Zhang L Chen C 2013 Exposure to bisphenol A disrupts meiotic progression during spermatogenesis in adult rats through estrogen-like activity. Cell Death Dis 4 e676; 10.1038/cddis.2013.203 23788033 PMC3702305

[r69] Machtinger R, Combelles CM, Missmer SA, Correia KF, Williams P, Hauser R (2013). Bisphenol-A and human oocyte maturation *in vitro*.. Hum Reprod.

[r70] Meeker JD, Calafat AM, Hauser R (2010a). Urinary bisphenol A concentrations in relation to serum thyroid and reproductive hormone levels in men from an infertility clinic.. Environ Sci Technol.

[r71] Meeker JD, Ehrlich S, Toth TL, Wright DL, Calafat AM, Trisini AT (2010b). Semen quality and sperm DNA damage in relation to urinary bisphenol A among men from an infertility clinic.. Reprod Toxicol.

[r72] Mendiola J Jørgensen N Andersson AM Calafat AM Ye X Redmon JB 2010 Are environmental levels of bisphenol A associated with reproductive function in fertile men? Environ Health Perspect 118 1286 1291; 10.1289/ehp.1002037 20494855 PMC2944091

[r73] Mendoza-Rodríguez CA, García-Guzmán M, Baranda-Avila N, Morimoto S, Perrot-Applanat M, Cerbón M (2011). Administration of bisphenol A to dams during perinatal period modifies molecular and morphological reproductive parameters of the offspring.. Reprod Toxicol.

[r74] Miao M, Yuan W, He Y, Zhou Z, Wang J, Gao E (2011a). In utero exposure to bisphenol-A and anogenital distance of male offspring.. Birth Defects Res A Clin Mol Teratol.

[r75] Miao M, Yuan W, Zhu G, He X, Li DK (2011b). *In utero* exposure to bisphenol-A and its effect on birth weight of offspring.. Reprod Toxicol.

[r76] Minamiyama Y, Ichikawa H, Takemura S, Kusunoki H, Naito Y, Yoshikawa T (2010). Generation of reactive oxygen species in sperms of rats as an earlier marker for evaluating the toxicity of endocrine-disrupting chemicals.. Free Radic Res.

[r77] Mok-Lin E, Ehrlich S, Williams PL, Petrozza J, Wright DL, Calafat AM (2010). Urinary bisphenol A concentrations and ovarian response among women undergoing IVF.. Int J Androl.

[r78] Morice L, Benaîtreau D, Dieudonné MN, Morvan C, Serazin V, de Mazancourt P (2011). Antiproliferative and proapoptotic effects of bisphenol A on human trophoblastic JEG-3 cells.. Reprod Toxicol.

[r79] Muhlhauser A Susiarjo M Rubio C Griswold J Gorence G Hassold T 2009 Bisphenol A effects on the growing mouse oocyte are influenced by diet. Biol Reprod 80 1067 1072; 10.1095/biolreprod.108.074815 PMC2804836 19164168

[r80] Nah WH, Park MJ, Gye MC (2011). Effects of early prepubertal exposure to bisphenol A on the onset of puberty, ovarian weights, and estrous cycle in female mice.. Clin Exp Reprod Med.

[r81] Nakamura D, Yanagiba Y, Duan Z, Ito Y, Okamura A, Asaeda N (2010). Bisphenol A may cause testosterone reduction by adversely affecting both testis and pituitary systems similar to estradiol.. Toxicol Lett.

[r82] Nanjappa MK, Simon L, Akingbemi BT. 2012 The industrial chemical bisphenol A (BPA) interferes with proliferative activity and development of steroidogenic capacity in rat Leydig cells. Biol Reprod 86 5 135, 1 12; 10.1095/biolreprod.111.095349 22302688 PMC3364919

[r83] Newbold RR Jefferson WN Padilla-Banks E 2009 Prenatal exposure to bisphenol A at environmentally relevant doses adversely affects the murine female reproductive tract later in life. Environ Health Perspect 117 879 885; 10.1289/ehp.0800045 19590677 PMC2702400

[r84] Nicholson TM, Ricke EA, Marker PC, Miano JM, Mayer RD, Timms BG (2012). Testosterone and 17β-estradiol induce glandular prostatic growth, bladder outlet obstruction, and voiding dysfunction in male mice.. Endocrinology.

[r85] Nicholson TM, Ricke WA (2011). Androgens and estrogens in benign prostatic hyperplasia: past, present and future.. Differentiation.

[r86] N’Tumba-Byn T Moison D Lacroix M Lecureuil C Lesage L Prud’homme SM 2013 Differential effects of bisphenol A and diethylstilbestrol on human, rat and mouse fetal Leydig cell function. PLoS One 7 12 e51579; 10.1371/journal.pone.0051579 23284716 PMC3524173

[r87] Okada A, Kai O (2008). Effects of estradiol-17beta and bisphenol A administered chronically to mice throughout pregnancy and lactation on the male pups’ reproductive system.. Asian J Androl.

[r88] Pacchierotti F, Ranaldi R, Eichenlaub-Ritter U, Attia S, Adler ID (2008). Evaluation of aneugenic effects of bisphenol A in somatic and germ cells of the mouse.. Mutat Res.

[r89] Padmanabhan V, Siefert K, Ransom S, Johnson T, Pinkerton J, Anderson L (2008). Maternal bisphenol-A levels at delivery: a looming problem?. J Perinatol.

[r90] Peretz J Craig ZR Flaws JA 2012 Bisphenol A inhibits follicle growth and induces atresia in cultured mouse antral follicles independently of the genomic estrogenic pathway. Biol Reprod 87 3 63; 10.1095/biolreprod.112.101899 22743301 PMC3464906

[r91] Peretz J, Gupta RK, Singh J, Hernandez-Ochoa I, Flaws JA (2011). Bisphenol A impairs follicle growth, inhibits steroidogenesis, and downregulates rate-limiting enzymes in the estradiol biosynthesis pathway.. Toxicol Sci.

[r92] Philippat C Mortamais M Chevrier C Petit C Calafat AM Ye X 2012 Exposure to phthalates and phenols during pregnancy and offspring size at birth. Environ Health Perspect 120 464 470; 10.1289/ehp.1103634 21900077 PMC3295340

[r93] Prins GS, Ye SH, Birch L, Ho SM, Kannan K (2011). Serum bisphenol A pharmacokinetics and prostate neoplastic responses following oral and subcutaneous exposures in neonatal Sprague-Dawley rats.. Reprod Toxicol.

[r94] Qiao L Zheng L Cai D 2010 Study on the levels of the bisphenol A, octylphenol, 4-nonylphenol in serum of precocious girls [in Chinese] Wei Sheng Yan Jiu 39 1 9 12 20364578

[r95] Qiu LL, Wang X, Zhang XH, Zhang Z, Gu J, Liu L (2013). Decreased androgen receptor expression may contribute to spermatogenesis failure in rats exposed to low concentration of bisphenol A.. Toxicol Lett.

[r96] Rashid H, Ahmad F, Rahman S, Ansari RA, Bhatia K, Kaur M (2009). Iron deficiency augments bisphenol A-induced oxidative stress in rats.. Toxicology.

[r97] Richter CA, Birnbaum LS, Farabollini F, Newbold RR, Rubin BS, Talsness CE (2007). *In vivo* effects of bisphenol A in laboratory rodent studies.. Reprod Toxicol.

[r98] Ricke WA, McPherson SJ, Bianco JJ, Cunha GR, Wang Y, Risbridger GP (2008). Prostatic hormonal carcinogenesis is mediated by *in situ* estrogen production and estrogen receptor alpha signaling.. FASEB J.

[r99] Rivera OE, Varayoud J, Rodriguez HA, Munoz-de-Toro M, Luque EH (2011). Neonatal exposure to bisphenol A or diethylstilbestrol alters the ovarian follicular dynamics in the lamb.. Reprod Toxicol.

[r100] Rodríguez HA, Santambrosio N, Santamaría CG, Muñoz-de-Toro M, Luque EH (2010). Neonatal exposure to bisphenol A reduces the pool of primordial follicles in the rat ovary.. Reprod Toxicol.

[r101] Ross C, Morriss A, Khairy M, Khalaf Y, Braude P, Coomarasamy A (2010). A systematic review of the effect of oral antioxidants on male infertility.. Reprod Biomed Online.

[r102] Ryan BC, Hotchkiss AK, Crofton KM, Gray LE (2010). *In utero* and lactational exposure to bisphenol A, in contrast to ethinyl estradiol, does not alter sexually dimorphic behavior, puberty, fertility, and anatomy of female LE rats.. Toxicol Sci.

[r103] Salian S, Doshi T, Vanage G (2009a). Neonatal exposure of male rats to bisphenol A impairs fertility and expression of Sertoli cell junctional proteins in the testis.. Toxicology.

[r104] Salian S, Doshi T, Vanage G (2009b). Perinatal exposure of rats to bisphenol A affects the fertility of male offspring.. Life Sci.

[r105] Sánchez P Castro B Torres JM Olmo A del Moral RG Ortega E 2013 Bisphenol A modifies the regulation exerted by testosterone on 5α-reductase isozymes in ventral prostate of adult rats. Biomed Res Int 2013 629235; 10.1155/2013/629235 PMC3741927 23984391

[r106] Signorile PG, Spugnini EP, Mita L, Mellone P, D’Avino A, Bianco M (2010). Pre-natal exposure of mice to bisphenol A elicits an endometriosis-like phenotype in female offspring.. Gen Comp Endocrinol.

[r107] Snijder CA Heederik D Pierik FH Hofman A Jaddoe VW Koch HM 2013 Fetal growth and prenatal exposure to bisphenol A: the generation R Study. Environ Health Perspect 121 393 398; 10.1289/ehp.1205296 23459363 PMC3621207

[r108] Susiarjo M Hassold TJ Freeman E Hunt PA 2007 Bisphenol A exposure in utero disrupts early oogenesis in the mouse. PLoS Genet 3 1 e5; 10.1371/journal.pgen.0030005 17222059 PMC1781485

[r109] Susiarjo M Sasson I Mesaros C Bartolomei MS 2013 Bisphenol A exposure disrupts genomic imprinting in the mouse. PLoS Genet 9 4 e1003401; 10.1371/journal.pgen.1003401 23593014 PMC3616904

[r110] Tainaka H, Takahashi H, Umezawa M, Tanaka H, Nishimune Y, Oshio S (2012). Evaluation of the testicular toxicity of prenatal exposure to bisphenol A based on microarray analysis combined with MeSH annotation.. J Toxicol Sci.

[r111] Tan W Huang H Wang Y Wong TY Wang CC Leung LK 2013 Bisphenol A differentially activates protein kinase C isoforms in murine placental tissue. Toxicol Appl Pharmacol 269 2 163 168; 10.1016/j.taap.2013.03.016 23545179

[r112] Tang WY, Morey LM, Cheung YY, Birch L, Prins GS, Ho SM (2012). Neonatal exposure to estradiol/bisphenol A alters promoter methylation and expression of *Nsbp1* and *Hpcal1* genes and transcriptional programs of *Dnmt3a/b* and *Mbd2/4* in the rat prostate gland throughout life.. Endocrinology.

[r113] Thuillier R, Manku G, Wang Y, Culty M (2009). Changes in MAPK pathway in neonatal and adult testis following fetal estrogen exposure and effects on rat testicular cells.. Microsc Res Tech.

[r114] Tiwari D Vanage G 2013 Mutagenic effect of bisphenol A on adult rat male germ cells and their fertility. Reprod Toxicol 40 60 68; 10.1016/j.reprotox.2013.05.013 23770294

[r115] Trapphoff T, Heiligentag M, El Hajj N, Haaf T, Eichenlaub-Ritter U. 2013 Chronic exposure to a low concentration of bisphenol A during follicle culture affects the epigenetic status of germinal vesicles and metaphase II oocytes. Fertil Steril 100 6 1758 1767.e1; 10.1016/j.fertnstert.2013.08.021 24034936

[r116] Tyl RW, Myers CB, Marr MC, Sloan CS, Castillo NP, Veselica MM (2008). Two-generation reproductive toxicity study of dietary bisphenol A in CD-1 (Swiss) mice.. Toxicol Sci.

[r117] U.S. EPA (U.S. Environmental Protection Agency). Bisphenol A (CASRN 80-05-7). Updated 07/01/1993.. http://www.epa.gov/iris/subst/0356.htm.

[r118] Vandenberg LN Chahoud I Heindel JJ Padmanabhan V Paumgartten FJ Schoenfelder G 2010 Urinary, circulating, and tissue biomonitoring studies indicate widespread exposure to bisphenol A. Environ Health Perspect 118 1055 1070; 10.1289/ehp.0901716 20338858 PMC2920080

[r119] Vandenberg LN, Ehrlich S, Belcher SM, Ben-Jonathan N, Dolinoy DC, Hugo ER, et al. 2013 Low dose effects of bisphenol A: An integrated review of *in vitro*, laboratory animal, and epidemiology studies. Endocr Disruptors 1 1e25078; 10.4161/endo.26490

[r120] Varayoud J, Ramos JG, Bosquiazzo VL, Lower M, Munoz-de-Toro M, Luque EH (2011). Neonatal exposure to bisphenol A alters rat uterine implantation-associated gene expression and reduces the number of implantation sites.. Endocrinology.

[r121] Varayoud J, Ramos JG, Bosquiazzo VL, Munoz-de-Toro M, Luque EH (2008). Developmental exposure to bisphenol A impairs the uterine response to ovarian steroids in the adult.. Endocrinology.

[r122] Veiga-Lopez A, Luense LJ, Christenson LK, Padmanabhan V (2013). Developmental programming: gestational bisphenol-A treatment alters trajectory of fetal ovarian gene expression.. Endocrinology..

[r123] vom Saal FS, Akingbemi BT, Belcher SM, Birnbaum LS, Crain DA, Eriksen M (2007). Chapel Hill bisphenol A expert panel consensus statement: integration of mechanisms, effects in animals and potential to impact human health at current levels of exposure.. Reprod Toxicol.

[r124] vom Saal FS VandeVoort C Taylor J Welshons W Toutain PL Hunt PA 2014 Bisphenol A (BPA) pharmacokinetics with daily oral bolus or continuous exposure via silastic capsules in pregnant rehesus monkeys:relevance for human exposures. Reprod Toxicol 45 105 116; 10.1016/j.reprotox.2014.01.007 24582107 PMC4035044

[r125] Wang Q, Zhao XF, Ji YL, Wang H, Liu P, Zhang C (2010). Mitochondrial signaling pathway is also involved in bisphenol A induced germ cell apoptosis in testes.. Toxicol Lett.

[r126] Wang Z, Liu BC, Lin GT, Lin CS, Lue TF, Willingham E (2007). Up-regulation of estrogen responsive genes in hypospadias: microarray analysis.. J Urol.

[r127] Willingham E, Baskin LS (2007). Candidate genes and their response to environmental agents in the etiology of hypospadias.. Nat Clin Pract Urol.

[r128] Wolff MS, Britton JA, Boguski L, Hochman S, Maloney N, Serra N (2008a). Environmental exposures and puberty in inner-city girls.. Environ Res.

[r129] Wolff MS Engel SM Berkowitz GS Ye X Silva MJ Zhu C 2008b Prenatal phenol and phthalate exposures and birth outcomes. Environ Health Perspect. 116 8 1092 1097; 10.1289/ehp.11007 18709157 PMC2516577

[r130] Wolff MS Teitelbaum SL Pinney SM Windham G Liao L Biro F 2010 Investigation of relationships between urinary biomarkers of phytoestrogens, phthalates, and phenols and pubertal stages in girls. Environ Health Perspect 118 1039 1046; 10.1289/ehp.0901690 20308033 PMC2920905

[r131] Wu HJ, Liu C, Duan WX, Xu SC, He MD, Chen CH (2013). Melatonin ameliorates bisphenol A-induced DNA damage in the germ cells of adult male rats.. Mutat Res.

[r132] Wu JH, Jiang XR, Liu GM, Liu XY, He GL, Sun ZY (2011). Oral exposure to low-dose bisphenol A aggravates testosterone-induced benign hyperplasia prostate in rats.. Toxicol Ind Health.

[r133] Xi W, Lee CK, Yeung WS, Giesy JP, Wong MH, Zhang X (2011). Effect of perinatal and postnatal bisphenol A exposure to the regulatory circuits at the hypothalamus– pituitary–gonadal axis of CD-1 mice.. Reprod Toxicol.

[r134] Xiao S, Diao H, Smith MA, Song X, Ye X (2011). Preimplantation exposure to bisphenol A (BPA) affects embryo transport, preimplantation embryo development, and uterine receptivity in mice.. Reprod Toxicol.

[r135] Yigit F, Daglioglu S (2010). Histological changes in the uterus of the hens after embryonic exposure to bisphenol A and diethylstilbestrol.. Protoplasma.

[r136] Zhang HQ, Zhang XF, Zhang LJ, Chao HH, Pan B, Feng YM (2012). Fetal exposure to bisphenol A affects the primordial follicle formation by inhibiting the meiotic progression of oocytes.. Mol Biol Rep.

[r137] Zhang XF, Zhang LJ, Feng YN, Chen B, Feng YM, Liang GJ (2012). Bisphenol A exposure modifies DNA methylation of imprint genes in mouse fetal germ cells.. Mol Biol Rep.

[r138] Zhou W, Liu J, Liao L, Han S, Liu J (2008). Effect of bisphenol A on steroid hormone production in rat ovarian theca-interstitial and granulosa cells.. Mol Cell Endocrinol.

[r139] Ziv-Gal A Craig ZR Wang W Flaws JA 2013 Bisphenol A inhibits cultured mouse ovarian follicle growth partially via the aryl hydrocarbon receptor signaling pathway. Reprod Toxicol 42 58 67; 10.1016/j.reprotox.2013.07.022 23928317 PMC3836856

