# Supplementary material for: Bisphenol A and Reproductive Health: Update of Experimental and Human Evidence, 2007–2013
Source: Environ Health Perspect. 2014 Jun 4;122(8):775–86. doi: 10.1289/ehp.1307728 (PMC4123031; doi:10.1289/ehp.1307728)
Supplement: (770 KB) PDF [file ehp.1307728.s001.pdf]

Environmental Health Perspectives doi:10.1289/ehp.1307728

Supplemental Material

Bisphenol A and Reproductive Health: Update of Experimental and Human Evidence, 2007–2013

Jackye Peretz, Lisa Vrooman, William A. Ricke, Patricia A. Hunt, Shelley Ehrlich, Russ Hauser, Vasantha Padmanabhan, Hugh S. Taylor, Shanna H. Swan, Catherine A. VandeVoort, and Jodi A. Flaws

Table of Contents **Page**

Table S1. BPA, ovarian and oviductal outcomes, and steroidogenesis in experimental studies **2**

Table S2. BPA and female human reproductive outcomes **6**

Table S3. BPA and uterine outcomes in experimental studies **10**

Table S4. BPA and human pregnancy and birth outcomes **13**

Table S5. BPA and pregnancy outcomes in experimental studies **16**

Table S6. BPA and male human reproductive outcomes **18**

Table S7. BPA and male reproductive outcomes in experimental studies **20**

Table S8. BPA and sexual function outcomes in experimental studies **25**

References **27**

Table S1. BPA, ovarian and oviductal outcomes, and steroidogenesis in experimental studies.

Source	Animal	Strain	Exposure route	Time of exposure	Doses	Age at collection	Outcome
Adewale et al. 2009	Rat	Long-Evans	Subcutaneous injection	Neonatal exposure: PNDO-PND3	50μg/kg and 50mg/kg	PND148	Disrupted ovarian development
Berger et al. 2008	Mouse	CF-1	Subcutaneous injection	Gestational exposure: GD1-GD4	0.01-300mg/kg	GD6	Decreased number of implantation sites, decreased progesterone
Berger et al. 2008	Mouse	CF-1	Subcutaneous injection	Gestational exposure: GD0,1, or 2	200 and 300mg/kg	GD6	Decreased number of implantation sites, decreased progesterone
Berger et al. 2010	Mouse	CF-1	Subcutaneous injection	Gestational exposure: GD1-GD4	100, 200, 300mg/kg	GD6	Altered estrogen receptor and progesterone gene expression
Brieño-Enriquez et al. 2011a	Human	NA	*In vitro*; Fetal oocytes	7, 14, 21 days	1, 5, 10, 20, 30μM	18-22 weeks old	Oocyte degeneration, impaired meiosis
Brieño-Enriquez et al. 2011b	Human	NA	*In vitro*; Fetal oocytes	7, 14, 21 days	30μM	18- 22 weeks old	Increased oocyte DNA double strand break, signaling, and repair genes
Chao et al. 2012	Mouse	CD-1	Subcutaneous injection	Neonatal exposure: PND7-PND14	20, 40μg/kg	PND15	Impaired methylation of imprinted genes during oogenesis, increases primordial follicular recruitment
Chao et al. 2012	Mouse	CD-1	Subcutaneous injection	Neonatal exposure: PND5,10,15,20	20, 40μg/kg	PND21	Impaired methylation of imprinted genes during oogenesis, increases primordial follicular recruitment
Eichenlaub-Ritter et al. 2008	Mouse	MF1	*In vitro*; Prepubertal oocytes	16 hours	0.22-43.8μM	After culture	Impaired meiosis, increased meiotic arrest, normal GVBD
Eichenlaub-Ritter et al. 2008	Mouse	MF1	Oral gavage	Neonatal exposure: PND22-PND28	20, 40, 100ng/g	PND28	Impaired meiosis, increased meiotic arrest, normal GVBD

Source	Animal	Strain	Exposure route	Time of exposure	Doses	Age at collection	Outcome
Fernandez et al. 2010	Rat	Sprague-Dawley	Subcutaneous injection	Neonatal exposure: PND1-PND10	5µg/50µL, 50µg/50µL, 500µg/50µL	4-5 months old	Increased testosterone and estradiol, increased follicular cysts, increased infertility
Grasseli et al. 2010	Pig	NA	*In vitro*, granulosa cells	48 hours	0, 0.1, 1, 10µM	NA	Altered steroidogenesis, increased VEGF
Hunt et al. 2012	Non-human primate	Rhesus macaque	Dietary exposure	Gestational exposure: GD50-GD100	400µg/kg	GD100	Impaired meiosis, increased multi-oocyte follicles, increased unenclosed and non-growing oocytes
Hunt et al. 2012	Non-human primate	Rhesus macaque	Dietary exposure	Gestational exposure: GD100-term	400µg/kg	PND0	Impaired meiosis, increased multi-oocyte follicles, increased unenclosed and non-growing oocytes
Hunt et al. 2012	Non-human primate	Rhesus macaque	Silastic pump	Gestational exposure: GD50-GD100	2.2-3.3ng/mL serum levels	GD100	Impaired meiosis, increased multi-oocyte follicles, increased unenclosed and non-growing oocytes
Hunt et al. 2012	Non-human primate	Rhesus macaque	Silastic pump	Gestational exposure: GD100-term	2.2-3.3ng/mL serum levels	PND0	Impaired meiosis, increased multi-oocyte follicles, increased unenclosed and non-growing oocytes
Kobayashi et al. 2012	Rat	Sprague-Dawley	Dietary exposure	Gestational and neonatal exposure: GD6-PND21	0, 0,33, 3,3, and 33 ppm	5 weeks or 3 months old	No change in hormones, AGD, lower ovarian weight
Lawson et al. 2011	Mouse	C57BL/6	Oral exposure	Gestational exposure: GD11-GD14.5	20ng/g	GD12, 12.5, 13.5, 14.5	Disrupted oogenesis via meiotic gene expression alterations
Lee SG et al. 2013	Rat	Sprague-Dawley	Oral gavage	Postnatal exposure: 90 days	0.001 and 0.1mg/kg	21 weeks old	Decreased testosterone and estradiol, increased atresia
Lenie et al. 2008	Mouse	C57BL/6j x CBA/Ca	*In vitro*: Early preantral follicles	12 days	3nM-30µM	NA	Impaired meiosis, impaired follicle development
Li Y et al. 2013	Rat	Wistar	Intraperitoneal injection	Neonatal exposure: 7 days	10, 40, 160mg/kg	35 days old	Decreased follicle numbers, increased atretic follicles

Source	Animal	Strain	Exposure route	Time of exposure	Doses	Age at collection	Outcome
Machtinger et al. 2013	Human	NA	*In vitro*: Adult oocytes	30 hours	20, 200ng/ml, 20µg/ml	Immature (GV stage) oocytes	Increased meiotic arrest, disrupted spindle formation and chromosome alignment, increased spontaneous oocyte activation
Mendoza-Rodriquez et al. 2011	Rat	Wistar	Drinking water	Gestational and neonatal exposure: GD6-PND21	10mg/L (1.2mg/kg)	7 weeks old	Increased uterine epithelial and stromal thickness, decreased uterine epithelial apoptosis, downregulation of *Esr1* expression in epithelial cells
Newbold et al. 2009	Mouse	CD-1	Subcutaneous injection	Gestational exposure: GD9-GD16	0, 0.1, 1, 10, 100, 1000µg/kg	18 months old	Induced ovarian cyst-adenomas, induced progressive proliferative oviductal lesions
Pacchierotti et al. 2008	Mouse	C57BL/6	Oral gavage	Postnatal exposure: Single dose	0.2 and 20mg/kg	4-11 weeks old	No effect on meiotic abnormalities
Pacchierotti et al. 2008	Mouse	C57BL/6	Oral gavage	Postnatal exposure: Single dose	0.04mg/kg	4-11 weeks old	No effect on meiotic abnormalities
Pacchierotti et al. 2008	Mouse	C57BL/6	Drinking water	Postnatal exposure: 7 weeks	0.5mg/L	4-11 weeks old	No effect on meiotic abnormalities
Peretz et al. 2012	Mouse	CD-1	*In vitro*:Antral follicles	24-96 hours	4.4-440µM	32-35 day old mice	Inhibited follicle growth, induced atresia, dysregulated cell cycle
Peretz et al. 2011	Mouse	FVB	*In vitro*:Antral follicles	24-120 hours	4.4-440µM	32-35 day old mice	Inhibited follicle growth, inhibited steroidogenesis
Rivera et al. 2011	Lamb	Hampshire Down	Subcutaneous injection	Neonatal exposure: PND1-PND14	50µg/kg	PND1, 5, 10, 30	Increased primordial follicular recruitment, increased multi-oocyte follicles, increased granulosa and theca cell proliferation, induced atresia
Rodríguez et al. 2010	Rat	Wistar	Subcutaneous injection	Neonatal exposure: PND1-PND7	0.05 and 20mg/kg	PND8	Increased primordial follicular recruitment, increased granulosa cell proliferation
Signorile et al. 2010	Mouse	Balb-c	Subcutaneous injection	Gestational exposure: GD1-PND7	100 and 1000µg/kg	3 months old	Induced endometriosis-like phenotype

Source	Animal	Strain	Exposure route	Time of exposure	Doses	Age at collection	Outcome
Tan et al. 2013	Mouse	ICR	Oral gavage	Gestational exposure: E13-E16	2, 20, 200mg/kg	E17	Increased estradiol, testosterone, and corticotropin releasing hormone
Trapphoff et al. 2013	Mouse	C57BL/6x CBA/Ca	*In vitro*: Pre-antral follicles	12 days	3 and 300nM	12-14 days old	Accelerated follicle development, disrupted genomic imprinting
Varayoud et al. 2011	Rat	Wistar	Subcutaneous injection	Neonatal exposure: PND1, 3, 5, and 7	0.05 and 20mg/kg	F1: GD5 or GD18	Down-regulated estrogen receptor and progesterone receptor
Veiga-Lopez et al. 2013	Lamb	Suffolk	Subcutaneous injection	Gestational exposure: GD30-GD90	0.5mg/kg	GD60 and GD90	Altered steroidogenic and miRNA gene expression
Xi et al. 2011	Mouse	CD-1	Oral gavage	Postnatal exposure, F0: GD1-PND20	12, 25, 50mg/kg	PND50	Disrupted feedback circuits of hypothalamic-pituitary-gonad axis
Xi et al. 2011	Mouse	CD-1	Oral gavage	Postnatal exposure, F1: PND21-PND49	12, 25, 50mg/kg	PND50	Disrupted feedback circuits of hypothalamic-pituitary-gonad axis
Xi et al. 2011	Mouse	CD-1	Oral gavage	Postnatal exposure, F1: PND21-PND49	25 and 50mg/kg	PND50	Disrupted feedback circuits of hypothalamic-pituitary-gonad axis
Zhang H et al. 2012	Mouse	CD-1	Oral exposure	Gestational exposure: GD12.5-PND18.5	0, 0.2, 0.04, 0.08mg/kg	GD15.5, 17.5, 19.5 PND3, 5, 7	Inhibits meiosis, impairs germ cell nest breakdown
Zhang X et al. 2012	Mouse	CD-1	Oral exposure	Gestational exposure: GD0.5-PND0	0, 40, 80, 160µg/kg	PND0	Altered methylation and expression of imprinting genes
Zhou et al. 2008	Rat	Sprague-Dawley	*In vitro:* Isolated follicular cells	72 hours	10^{-4} to 10^{-7}M	30 days old	Altered steroidogenic enzyme expression and steroidogenesis
Ziv-Gal et al. 2013	Mouse	C57BL/6	*In vitro*: Antral follicles	96 hours	0.004 to 438µM	54-58 days old	Inhibited follicle growth, inhibited estradiol levels

AGD=anogenital distance; ESR1=estrogen receptor 1; F1=first filial generation; GD=gestation day; GV=germinal vesicle; GVBD=germinal vesicle breakdown; NA=not applicable; PND=postnatal day

Table S2. BPA and female human reproductive outcomes.

Source	Study design	Study population	Sample size	Timing of BPA measurement	BPA concentration (mean, geometric mean or median)	Outcome	Comments/limitations
Bloom et al. 2011a	Cross-sectional	Women undergoing IVF (University of California-San Francisco)	44	Preconception (day of egg retrieval)	Median: 2.53ng/mL serum (unconjugated)	Negative association of BPA with peak estradiol (β -0.16, 95% CI: -0.32, 0.01); null association with # oocytes retrieved (aRR: 0.95, 95% CI: 0.827, 1.10)	Small sample size, BPA measured once per treatment cycle
Bloom et al. 2011b	Prospective cohort	Couples undergoing IVF (University of California-San Francisco)	27 couples (36 women)	Preconception (day of egg retrieval)	Median: 3.3ng/ml (women) 85% detection rate vs. 0.48ng/mL (men) 53% detection rate, serum (unconjugated)	Negative association between male BPA and embryo quality (aOR: 0.54, p = 0.009) and normal embryo cleavage rate (aOR: 0.70, p = 0.07); null association of female BPA and embryo quality	Small sample size, BPA measured once, larger than expected difference in median serum BPA in men and women
Buck Louis et al. 2013	Matched cohort	Women undergoing laparoscopy/laparomy	495 individuals in operative cohort and 131 women in population cohort	At the time of laparoscopy/ screeing for endometriosis	Geometric mean for operative cohort: 1.45 (1.14-1.84) ng/ml for women with endometriosis and 1.66 (1.40-1.97) ng/ml for women without endometriosis; geometric mean for population cohort: 4.19 (2.18-8.03) ng/ml for women with endometriosis and 1.65 (1.23-2.23) ng/ml for women without endometriosis	Null association between BPA exposure and endometriosis (aOR: 0.96, 95% CI: 0.79, 1.19 for operative cohort; aOR: 1.68, 95% CI: 0.96, 2.92 for population cohort	Collection of urine samples across the menstrual cycle, not originally powered to examine BPA and endometriosis, short interval between measurement of BPA and diagnosis of endometriosis, exploratory analysis
Cobellis et al. 2009	Case-control	Women undergoing laparoscopy for chronic pelvic pain	69 (58 cases, 11 controls)	At the time of laparoscopy/ diagnosis of endometriosis	Mean± SD: 2.91 ± 1.74 ng/mL serum (in a subset of 15 women)	Positive association of BPA with endometriosis. BPA was detectable in 52% of women with endometriosis. No detectable BPA in controls.	Small sample size, limited information on exposure and confounders

Source	Study design	Study population	Sample size	Timing of BPA measurement	BPA concentration (mean, geometric mean or median)	Outcome	Comments/limitations
Ehrlich et al 2012a	Prospective cohort	Women undergoing IVF (Massachusetts General Hospital)	137 (180 IVF cycles)	Preconception (day of egg retrieval and start of cycle)	Median: 1.53 ng/mL urine (specific gravity-adjusted)	Positive linear dose response association between BPA and implantation failure (aOR: 1.02, 1.60 and 2.11 for BPA quartiles 2, 3 and 4 compared to quartile 1 (p-trend = 0.06)	Not adjusted for male BPA exposure or semen quality, BPA measured twice per treatment cycle
Ehrlich et al. 2012b	Prospective cohort	Women undergoing IVF (Massachusetts General Hospital)	174 (237 IVF cycles)	Preconception (day of egg retrieval and start of cycle)	Median: 1.50ng/mL urine (specific gravity-adjusted)	Negative association of female BPA with peak estradiol: mean decrease of 40, 253 and 471 pg/ml (p-trend 0.001); mean number of retrieved oocytes, mature and normally fertilized oocytes decreased by 24% to 27% respectively for highest vs. lowest BPA quartile (p-trend = 0.002); trend in decreased blastocyst formation (p-trend = 0.08)	Not adjusted for male BPA exposure or semen quality, BPA measured twice per treatment cycle
Ehrlich et al. 2013	Subset of ongoing prospective cohort	Women undergoing IVF (Massachusetts General Hospital)	61 women (76 IVF cycles)	Preconception (day of egg retrieval and start of cycle)	Median: 2.59 ug/L for early cycle (day 3-9) and 1.97 ug/L for the retrieval day urine	Possible U-shaped association of BPA with mean Cyp19 expression: β estimate (95% CI) for quartiles 2, 3, and 4 compared to lowest quartile: -0.97 (-2.22, 0.28), -0.97 (-2.18, 0.24), and -0.38 (-1.58, 0.82)	Limited sample size, unknown if urinary BPA levels reflects BPA levels at the ovarian follicle, timing of urine sample may not reflect relevant biological window of exposure

Source	Study design	Study population	Sample size	Timing of BPA measurement	BPA concentration (mean, geometric mean or median)	Outcome	Comments/limitations
Fujimoto et al. 2011	Cohort	Women undergoing ICSI (University of California-San Francisco)	58 infertile female and 37 male partners undergoing IVF	Preconception (day of egg retrieval)	Median: 2.53 ng/mL (women); 0.34 ng/mL (men) serum (unconjugated)	Null association between female BPA and oocyte maturation (aRR: 1.01, 95% CI: 0.98, 1.05). In 9 Asian women, significantly decreased oocyte maturation for a doubling of BPA (aRR: 0.91, 95% CI: 0.83, 1.00). Negative association of female BPA with fertilization (aRR: 0.45, 95% CI 0.21, 0.66)	Small sample size, single serum BPA, timing of male exposure not clear, larger than expected difference in median serum BPA in men and women
Galloway et al. 2010	Cross-sectional	Premenopausal women (INChianti cohort, Italy)	61	Random day of menstrual cycle	Geometric mean: 3.25 ng/mL (3.04–3.47) urine (creatinine-adjusted)	Null association of BPA with estradiol (β = - 0.026, 95% CI: -0.066, 0.014), null association with total testosterone; positive association with SHBG (β = 0.029, 95% CI: 0.004, 0.054)	Small sample size, single urine BPA estradiol measured at non-specified time during menstrual cycle
Kandaraki et al. 2011	Case-control	PCOS clinic cases and gynecology controls (Greece)	171 (71 cases PCOS; 100 controls)	At time of diagnosis of PCOS, follicular phase	Mean ± SD: 1.05±0.56 ng/mL (PCOS) vs. 0.72±0.37 ng/mL [controls (p value<0.001)] serum	Positive association of BPA with PCOS (r = 0.497, p <0.05) for all PCOS cases vs. controls and also after stratifying by lean and obese phenotypes	Cases and controls matched by age and BMI, serum BPA by ELISA (sub-optimal)
Lee SH et al. 2013	Case-control	Girls experiencing precocious puberty	114 (82 cases; 32 controls)	Childhood (7-10yr at regular checkup)	Mean ± SD: peripheral-precocious puberty, 8.7 ± 7.6µg/g creatinine; central-precocious puberty, 8.0 ± 9.9µg/g creatinine; controls, 6.6 ± 7.3µg/g creatinine urine	Null association of BPA with precocious puberty. No differences in steroid metabolism between girls with and without precocious puberty, but possible positive association between BPA levels and testosterone, estradiol and pregnenolone in all subjects.	Cases and controls matched by age, urine BPA by GC-MS, small sample size

Source	Study design	Study population	Sample size	Timing of BPA measurement	BPA concentration (mean, geometric mean or median)	Outcome	Comments/limitations
Mok-Lin et al. 2010	Prospective cohort	Women undergoing IVF (Massachusetts General Hospital)	84 (112 IVF cycles)	Preconception (day of egg retrieval and start of cycle)	Median: 2.53ng/mL urine (specific gravity-adjusted)	Negative association of BPA with peak estradiol and oocyte yield with 213pg/ml ($p = 0.03$) decrease in estradiol, and 12% ($p = 0.007$) decrease in number of oocytes retrieved per log unit increase in BPA	Small sample size, no adjustment for male exposure or semen quality, BPA measured twice per treatment cycle
Qiao et al. 2010	Case-control	Girls with and without precocious puberty	110 girls with precocious puberty and 100 girls without precocious puberty	At time of estradiol, ovary, and uterine measurements	Detected in 40.9% of precocious girls and 2% of controls	Positive association with volume of the uterus ($r = 0.557$, $p < 0.05$) and volume of the ovary ($r = 0.469$, $p < 0.01$) in precocious girls	Small sample size, no longitudinal data
Wolff et al. 2008a	Cross-sectional	9 year old girls (inner city New York city 1996-1997)	192	At time of pubertal assessment in childhood	Geometric mean: 0.24 vs. 0.11µg/g creatinine for breast stage 1 vs. 2; 0.19 vs. 0.10 µg/g creatinine for pubic hair stage 1 vs. 2	Null association of BPA with breast development (PR: 0.96, 95% CI: 0.92, 1.01) and pubic hair growth (PR: 0.98, 95% CI: 0.89, 1.08)	Cross-sectional study
Wolff et al. 2008b	Prospective cohort	Mother-infant pairs	404	Mainly third trimester of pregnancy (25% of samples collected between 25-30 weeks, 45% between 31-35 weeks, and remainder between 36-40 weeks)	Range : 0.7-35.2 µL	Null association of BPA with birth weight, birth length, head circumference, and gestational age	One measure of BPA during third trimester
Wolff et al. 2010	Prospective cohort	6-8 year old girls (multicenter: NYC, Cincinnati, California, 2004-2007)	1151	Childhood (6-8yr at baseline visit)	Median : 2.0 ng/ mL urine (creatinine-adjusted)	Null association of BPA with early onset of pubertal development (p-trend = 0.53)	One measure of BPA at baseline –1 year before reassessment of puberty development

aOR=adjusted odds ratio; aRR=adjusted relative risk; CI=confidence interval; ELISA=enzyme-linked immunoabsorbant assay; GC-MS=gas chromatography-mass spectrometry; ICSI= intracytoplasmic sperm injection; IVF=*in vitro* fertilization; PCOS=polycystic ovarian syndrome

Table S3. BPA and uterine outcomes in experimental studies.

Source	Animal	Strain	Exposure route	Time of exposure	Doses	Age at collection	Outcome
Aghajanova et al. 2011	Human	NA	*In vitro*: Endometrial stromal fibroblasts	48 hours	5-100μmol/L	NA	Decreased steroidogenic gene expression
Aldad et al. 2011	Non-human primate	African green monkey	Alzet minipump	Postnatal exposure: 28 days	50μg/kg	Adult	Decreased progesterone receptor expression
Aldad et al. 2011	Non-human primate	African green monkey	*In vitro*: Ishikawa cells	24 hours	1μM	Adult	Decreased progesterone receptor expression
An et al. 2013	Rat	Sprague-Dawley	Subcutaneous injection	Neonatal exposure: PND17-PND19	10, 100, 500mg/kg	PND20	Decreased contractile activity
Benachour and Aris 2009	Human	NA	*In vitro*: Cytotrophoblasts	24 hours	0.0002- 200μg/mL	After 24h of culture	Increased apoptosis and necrosis, induced AK activity and *Tnfα* gene and protein expression
Berger et al. 2008	Mouse	CF-1	Subcutaneous injection	Gestational exposure: GD1-GD4	0.01-300mg/kg	GD6	Decreased number of implantation sites, decreased progesterone
Berger et al. 2008	Mouse	CF-1	Subcutaneous injection	Gestational exposure: GD0,1, or 2	200 and 300mg/kg	GD6	Decreased number of implantation sites, decreased progesterone
Berger et al. 2010	Mouse	CF-1	Subcutaneous injection	Gestational exposure: GD1-GD4	100, 200, 300mg/kg	GD6	Increased luminal epithelial cell height, altered estrogen receptor and progesterone receptor gene expression
Bosquiazzo et al. 2010	Rat	Wistar	Subcutaneous injection	Neonatal exposure: PND1,3,5,7	0.05 and 20mg/kg	PND94	Decreased endometrial proliferation, decreased *Vegf* expression
Bredhult et al. 2009	Human		*In vitro*: Endometrial epithelial cells	24 hours	50μM	After 24h of culture	Decreased endometrial epithelial cell proliferation
Bromer et al. 2010	Mouse	CD-1	Intraperitoneal injection	Gestational exposure: GD9-GD16	5mg/kg	2 or 6 weeks old	Decreased methylation of *Hoxa10* promoter

Source	Animal	Strain	Exposure route	Time of exposure	Doses	Age at collection	Outcome
Hiyama et al. 2011	mouse	ICR	Subcutanous injection	Gestational exposure: GD12-GD16	100-1000mg/kg	F1 and F2: 8 weeks	Increased uterine luminal space. decreased uterine epithelium, decreased methylation of *Hoxa10* intron
Mendoza-Rodriquez et al. 2011	Rat	Wistar	Drinking water	Gestational and neonatal exposure: GD6-PND21	10mg/L (1.2mg/kg)	7 weeks old	Increased uterine stroma and epithelium thickness, decreased uterine epithelial apoptosis
Morice et al. 2011	Human	NA	*In vitro*: JEG-3 trophoblast cells	24-72h	10^{-5}M to 10^{-10}M	4-8 week placental cells	Decreased cell proliferation. Increased apoptosis
Newbold et al. 2009	Mouse	CD-1	Subcutaneous injection	Gestational exposure : GD9-16	0, 0.1, 1, 10, 100, 1000μg/kg	18months old	Increased uterine abnormalities
Salian et al. 2009a	Rat	Holtzman	Subcutaneous injection	Neonatal exposure (males only): PND1-PND5	100-1600μg/kg	PND15, 30, 45, 90	Increased fetal resorption, increased pre-implantation loss, decreased litter size
Salian et al. 2009b	Rat	Holtzman	Oral gavage	Gestational and neonatal exposure (males only): GD12-PND21	1.2 and 2.4μg/kg	GD20 or PND125	Increased fetal resorption, decreased litter size, increased hormone imbalances
Signorile et al. 2010	Mouse	Balb-c	Subcutaneous injection	Gestational exposure : GD1-PND7	100 and 1000μg/kg	3 months old	Increased endometriosis-like structures
Susiarjo et al. 2013	Mouse	C57BL/6 Cast7	Dietary exposure	Gestational exposure: Prebreed to GD9.5	10μg/kg and 10mg/kg	F1: GD9.5	Disrupted imprinted gene expression in embryos and placenta
Tan et al. 2013	Mouse	ICR	Oral gavage	Gestational exposure : GD13-GD16	2, 20, 200mg/kg	GD17	Activated protein kinase C signaling
Tiwari and Vanage 2013	Rat	Holtzman	Oral gavage	Postnatal exposure (males only): 6 days	5 and 10μg/kg	GD15 (untreated female mated to treated male)	Decreased implantation sites, increased resorption sites

Source	Animal	Strain	Exposure route	Time of exposure	Doses	Age at collection	Outcome
Varayoud et al. 2008	Rat	Wistar	Subcutaneous injection	Neonatal exposure: PND 1, 3, 5, and 7	0.5 and 20mg/kg	PND8 or PND94	Decreased steroid hormone responsiveness of uterine stroma, decreased *Hoxa10* and *Hoxa11* expression
Varayoud et al. 2011	Rat	Wistar	Subcutaneous injection	Neonatal exposure: PND1, 3, 5, and 7	0.05 and 20mg/kg	F1: GD5 or GD18	Decreased implantation sites, decreased implantation-regulating gene expression
Xiao et al. 2011	Mouse	C57BL/6	Subcutaneous injection	Gestational exposure: GD0.5-GD3.5	0, 0.025, 0.5, 10, 40, 100mg/kg	GD4.5 or GD5.5	Ablated implantation, decreased embryo transport
Yigit and Daglioglu 2010	Chicken	White Leghorn	In ovo injection	Incubation day 4	67 and 134µg/g	21 weeks old	Decreased hatching proportion, uterine tubular glandular density, and thickness of tunica mucosa

AK=adenylate kinase; GD=gestation day; HOXA=homeobox A; GD=gestation day; PND=postnatal day; Tnfa=tumor necrosis factor alpha; vegf=vascular endothelial factor

Table S4. BPA and human pregnancy and birth outcomes.

Source	Study design	Study population	Sample Size	Timing of BPA measurement	BPA concentration (mean, geometric mean or median)	Outcome	Comments/limitations
Cantowine et al. 2010	Nested case-control in a prospective cohort	Pregnant women Mexico city (ELEMENT cohort)	60 (12(20%) preterm births)	Third trimester archived spot urine samples	Geometric mean: 1.45ng/mL for all women; 1.94ng/mL for women delivering preterm. Urine (SG- adjusted)	Borderline positive BPA association with preterm birth, (OR 1.91, 95% CI: 0.93, 3.91)	Pilot study, small sample size
Chou et al. 2011	Cross-sectional	Taiwanese mothers and infants	97	At delivery	Geometric mean: 2.5ng/mL (maternal serum); 0.5ng/mL (neonatal cord blood)	Positive association of BPA with low birth weight (OR 2.42, 95% CI: 1.72, 3.36); small for gestational age (OR 2.01, 95% CI: 1.39, 3.01); high leptin (OR 1.67, 95% CI: 1.12, 2.25); and low adiponectin (OR 1.25, 95% CI: 1.52, 3.97)	Small sample size, cross sectional design
Fenichel et al. 2012	Matched case-control	Boys with cryptorchidism (France)	152 (46 cases; 106 controls)	Cord blood at birth	Median: 0.86ng/mL in controls); 0.92ng/mL in cases serum (unconjugated)	Null association of BPA with cryptorchidism. However, significant positive correlation with testosterone and inhibin B levels	Exposure assessment of maternal BPA during pregnancy is important given the short half -life of BPA
Lee BE et al. 2013	Multi-center birth cohort	Korean mothers and children	757	Third trimester	Geometric mean: 1.29μg/L (1.87μg/g creatinine) during late pregnancy	Positive association between BPA levels and birth weight; second tertile of maternal BPA exposure exhibited an increase in birth weight relative to the first tertile ($p = 0.04$)	Urine levels of BPA may not reflect circulating levels, used spot urines. BPA values normalized to creatinine only
Miao et al. 2011a	Occupational cohort retrospective	Children of occupationally exposed Chinese parents	587	Exposure history and personal air sampling, job/exposure matrix; current urine in a subgroup, but not for index pregnancy	Geometric mean: Exposed mothers (direct exposure): 15.98μg/g Cr (N=50). Spouses of exposed men (indirect): 2.22μg/g Cr (N=93). Unexposed mothers: 0.56μg/g Cr (N=444) urine (cr-adjusted)	Negative association of BPA with birth weight (β = -168g, $p = 0.02$) for directly exposed fetus (maternal exposure); (β = -91g, p = 0.10) for indirectly exposed fetus (father exposed during pregnancy)	30-50% of women had their index pregnancy over 15 years prior to enrollment to the study, exposure was partly estimated using exposure history of occupational exposure, self-report of baby's birth weight. possible recall bias exists due elapsed time

Source	Study design	Study population	Sample Size	Timing of BPA measurement	BPA concentration (mean, geometric mean or median)	Outcome	Comments/limitations
Miao et al. 2011b	Occupational cohort study retrospective	Children of occupationally exposed parents (age 0-17 years)	153 (106 controls, 46 cases)	Personal air sampling at time of index pregnancy, current urine for different categories of exposure as defined using occupational history and job/exposure matrix	Geometric mean: Exposed mothers (direct exposure): 16.0 µg/g Cr (N=18). Spouses of exposed men (indirect): 2.2µg/g Cr (N=38). Unexposed mothers: 0.6µg/g Cr(N=97) Urine (cr-adjusted)	Positive linear dose response association of BPA with shortened AGD in boys by level of exposure, p-trend = 0.008; in boys of exposed mothers, decrease in AGD of 8.11mm (p = 0.003) and 2.87mm (p = 0.15) in boys of exposed fathers during index pregnancy	Wide age range of children at time of AGD measurement. Almost 20% of boys were older than 10 years. Uncertain generalizability to the environmentally exposed population
Padmanabhan et al. 2008	Cross-sectional	Southeastern Michigan mothers	40	At delivery	Mean (SEM): 5.9 (0.94) ng/mL serum (unconjugated)	Null association of BPA with birth weight and gestational length. Mothers with maternal BPA concentrations less than or equal to 5ng/ml had babies with similar birth weight to mothers with maternal BPA concentrations above 5ng/mL	Small sample size, limited information on exposure by birthweight (cut-off of 5ng/mL used in analyses), single spot serum at delivery
Philippat et al. 2012	Nested case-control	Mother-infant male pairs (France)	191	6-30 weeks gestation	Median: 3.1ng/mL (in mother) urine	Suggestive inverted U-shape association between BPA and birth weight in tertile analysis 169g (95% CI: 14, 324); 85g (-62, 233) for tertile 2 and 3, respectively. Increase in head circumference by 0.3cm (95% CI: 0.0, 0.7)	Single spot urine sample at one time point during pregnancy
Snijder et al. 2013	Prospective cohort study	Pregnant Dutch women	219	Early, mid, and late pregnancy	Geometric mean (SEM): 3.2 (2.3) µg/g creatinine	Negative association of BPA with fetal growth and head circumference. Among 80 women with three BPA measurements, women with BPA levels above 4.22µg/g creatinine had lower fetal growth rates and fetuses with smaller head circumference than women with BPA levels less than 1.54µg/g creatinine.	Small sample size, difficult to obtain solid estimate of first trimester fetal growth

Source	Study design	Study population	Sample Size	Timing of BPA measurement	BPA concentration (mean, geometric mean or median)	Outcome	Comments/limitations
Wolff et al. 2008b	Prospective cohort	Mother-infant pairs	404	Mainly third trimester of pregnancy (25% of samples collected between 25-30 weeks, 45% between 31-35 weeks, and remainder between 36-40 weeks)	Range : 0.7-35.2μL	Null association of BPA with birth weight, birth length, head circumference, and gestational age	One measure of BPA during third trimester

AGD=anogenital distance; CI=confidence interval; Cr=creatinine; OR=odds ratio; SEM=standard error of the mean; SG=specific gravity

Table S5. BPA and pregnancy outcomes in experimental studies.

Source	Animal	Strain	Exposure route	Time of exposure	Doses	Age at collection	Outcome
Cabaton et al. 2011	Mouse	CD-1	Alzet pump	Gestational exposure: GD8-PND16	25ng, 250ng, 25µg/kg	2-8months old	Decreased number of pregnancies, decreased number of pups
Howdeshell et al. 2008	Rat	Long-Evans	Oral gavage	Gestational exposure: GD7-PND18	2, 20, 200µg/kg	PND150	No adverse effects on pregnancy outcomes
Kobayashi et al. 2010	Mouse	C57BL/6	Dietary exposure	Gestational, neonatal, and postnatal exposure: F1: GD6-PND22; postnatal until sacrifice	0.05, 0.5, 5.0mg/kg	F1: 13 weeks	No adverse effects on pregnancy outcomes
Kobayashi et al. 2010	Mouse	C57BL/6	Dietary exposure	Gestational, neonatal, and postnatal exposure: F2: GD6-PND22; postnatal until sacrifice	0.05, 0.5, 5.0mg/kg	F2:15 weeks	No adverse effects on pregnancy outcomes
Kobayashi et al. 2012	Rat	Sprague-Dawley	Dietary exposure	Gestational and neonatal exposure: GD6-PND21	0.05, 0.5, 5.0mg/kg	5weeks; 3 months	No adverse effects on pregnancy outcomes
Nah et al. 2011	Mouse	ICR	Subcutaneous injection	Neonatal exposure: PND8	0.1, 1, 10, 100mg/kg	PND20-PND29; PND25, 30, 70	Decreased pup weight
Nanjappa et al. 2012	Rat	Long-Evans	Oral gavage	Gestational exposure: GD12-PND21	2.5 and 25µg/kg	PND90	No adverse effects on pregnancy outcomes
Ryan BC et al. 2010	Rat	Long-Evans	Oral gavage	Gestational and neonatal exposure: GD7-PND18	2, 20, 200µg/kg		No adverse effects on pregnancy outcomes
Salian et al. 2009a	Rat	Holtzman	Subcutaneous injection	Neonatal exposure (males only): PND1-5	100-1600µg/kg	PND15, 30, 45, 90	Decreased number of pups
Salian et al. 2009b	Rat	Holtzman	Oral gavage	Gestational exposure (males only): GD12-PND21	1.2 and 2.4µg/kg	Males: PND125 Females: GD20	Decreased number of pups
Tan et al. 2013	Mouse	ICR	Oral gavage	Gestational exposure: GD13-GD16	2, 20, 200mg/kg	GD17	Placenta gene changes that could cause pre-term birth
Thuillier et al. 2009	Rat	Sprague-Dawley	Oral gavage	Gestational exposure: GD14-PND0	0.1 to 200mg/kg	PND 3, 21, 60	No adverse effects on pregnancy outcomes
Tyl et al. 2008	Mouse	CD-1	Dietary exposure	Gestational exposure: F0 8 weeks pre-breed, GD1-GD14	0.003, 0.03, 0.3, 5, 50, 600mg/kg	PND1/birth	Increased pup weight

Source	Animal	Strain	Exposure route	Time of exposure	Doses	Age at collection	Outcome
Tyl et al. 2008	Mouse	CD-1	Dietary exposure	Postnatal exposure: F1:8 weeks pre-breed	0.003, 0.03, 0.3, 5, 50, 600mg/kg	PND1/birth	Increased pup weight
Xi et al. 2011	Mouse	CD-1	Oral gavage	Postnatal exposure: F0: GD1-PND20	12, 25, 50mg/kg	PND50	No adverse effects on pregnancy outcomes
Xi et al. 2011	Mouse	CD-1	Oral gavage	Postnatal exposure: F1: PND21-PND49	12, 25, 50mg/kg	PND50	No adverse effects on pregnancy outcomes
Xi et al. 2011	Mouse	CD-1	Oral gavage	Postnatal exposure: F1: PND21-49	12, 25, 50mg/kg	PND50	No adverse effects on pregnancy outcomes
Yigit and Daglioglu 2010	Chicken	White Leghorn	In ovo injection	Incubation day 4	67 and 134µg/g	21 weeks old	Decreased hatching

F0=parental generation; F1=first filial generation; GD=gestation day; PND=postnatal day

Table S6. BPA and male human reproductive outcomes.

Source	Study design	Study population	Sample Size	Timing of BPA measurement	BPA concentration (mean, geometric mean or median)	Outcome	Comments/limitations
Galloway et al. 2010	Cross-sectional	Italian adults between 20-74 years of age (INChianti)	715	Adulthood, same day as testosterone measure	Geometric mean: 4.02ng/mL (3.76–4.31) urine (creatinine-adjusted)	Positive association of BPA with total testosterone (β = 0.05, 95% CI: 0.02, 0.08), no association with sex hormone binding globulin and free testosterone	Relatively high BPA concentration for general population, cross-sectional study design, spot urine sample
Li DK et al. 2010	Cross-sectional	Occupationally exposed men from 4 regions of China	427	Pre and post shift	Median : 53.7µg/g creatinine (occupationally exposed) and 1.2µg/g Cr (unexposed) urine (creatinine-adjusted)	Positive association of BPA with sexual dysfunction, decreased sexual desire (β = 0.016, p < 0.001), erectile dysfunction (β = 0.022, p < 0.001), orgasmic function (β = -0.017, p < 0.001), and overall satisfaction with sex life (β = -0.010, p = 0.003)	Self-reported sexual dysfunction, low participation rate, possible current co-exposures
Li DK et al. 2011	Prospective cohort study	Occupationally exposed Chinese men	218	Pre and post shift	Median: 38.7ng/mL (occupationally exposed) and 1.4ng/mL (unexposed) urine (creatinine-adjusted)	Negative linear association of BPA with sperm concentration (β = 15.6, p < 0.001); sperm count (β = -42.1, p = 0.004) and increased odds of decreased sperm motility (OR: 2.3, 95% CI: 1.0, 5.1). Associations stronger after excluding occupationally exposed men.	Only two semen samples collected at 1-3 week intervals, small sample size for sub-analysis on men not occupationally exposed to BPA (N=88), uncertain generalizability to men exposed to environmentally relevant BPA concentrations
Meeker et al. 2010a	Cross-sectional	Male partners of couples seeking treatment for subfertility (Massachusetts General Hospital)	190	Same day as semen sample collection (additional urine samples for a subgroup collected weeks to months prior to semen sample)	Median: 1.3ng/mL urine (specific gravity-adjusted)	Negative association of BPA with sperm concentration: -23% (95% CI: -40%, -0.3%), morphology: -13% (95% CI: -26%, -0.1%), suggestive decrease in sperm motility: -7.5% (95% CI: -17%, 1.5%), and increase in sperm DNA damage: 10% (95% CI: 0.03%, 19%)	Associations only observed when semen parameters were modeled on a continuous scale, but not when modeled as binary outcome, uncertain generalizability of results to men from general population

Source	Study design	Study population	Sample Size	Timing of BPA measurement	BPA concentration (mean, geometric mean or median)	Outcome	Comments/limitations
Meeker et al. 2010b	Cross-sectional	Male partners of couples seeking treatment for subfertility (Massachusetts General Hospital)	167	Same day as blood sample for hormone measurements. Repeat samples in 75 men up to 2.5 months later	Median: 1.3ng/mL urine (specific gravity-adjusted)	Negative association of BPA with estradiol:testosterone ratio (β = 0.86, p = 0.01) and free androgen index (β = 0.89, p = 0.02), and positively associated with FSH (β = 1.18, p = 0.01) and FSH:inhibin B ratio (β = 1.28, p = 0.05)	Cross sectional study design, megative association with estradiol:testosterone ratio was consistent when modeling repeat urine BPA concentrations, but only a limited number of repeat samples were available, generalizability to men of general population is uncertain
Mendiola et al. 2010	Cross-sectional	Fertile male partners of pregnant women (Multicenter: Study for Future Families)	302 men (hormones) 317 men (semen quality)	Same day as hormone profile and semen collection	Median: 1.7 ng/mL urine (unadjusted) creatinine-adjusted	Positive association of BPA with sex hormone binding globulin (β = 0.07, 95% CI: 0.007, 0.13). Negative association with free androgen index (β = -0.01, 95% CI: -0.09, -0.004) and free androgen index: luteinizing hormone ratio (β = -0.11, 95% CI: -0.18, -0.03). Null associations with semen quality parameters.	Cross sectional study design

CI=confidence interval; Cr= creatinine; FSH=follicle-stimulating hormone; OR=odds ratio

Table S7. BPA and male reproductive outcomes in experimental studies.

Source	Animal	Strain	Exposure route	Time of exposure	Doses	Age at collection	Outcome
Arase et al. 2011	Mouse	C57BL/6	Oral gavage	Gestational exposure: GD13-GD16	20µg/kg	GD17 to PND1	Increased estradiol levels, increased steroidogenic enzyme expression in urogenital sinus
Anjum et al. 2013	Mouse	Swiss albino	Oral exposure	Postnatal exposure: adult	10mg/kg body weight	Adult, exact age unknown, after 14 days of dosing	Increased oxidative stress in testes
Castro et al. 2013	Rat	Wistar	Subcutaneous injection	Postnatal exposure: adult (4 days)	25, 50, 300, and 600µg/kg	Adult (exact age not reported, 30 min after last injection)	Decreased testosterone, increased estradiol
D'Cruz et al. 2012a	Rat	Wistar	Oral gavage	Postnatal exposure: 90-135 days old (45 days)	0.005, 0.5, 50, 500µg/kg	136 days old	Decreased expression of glucose transporters, increased reactive oxygen species production
D'Cruz et al. 2012b	Rat	Wistar	Oral gavage	Postnatal exposure: 90-135 days old (45 days)	0.005, 0.5, 50, 500µg/kg	136 days old	Decreased insulin signaling, decreased glucose transport, decreased steroidogenic enzymes. decreased testosterone
De Flora et al. 2011	Rat	Sprague-Dawley	Drinking water	Postnatal exposure: adult (10 days)	200mg/kg	Adult (exact age not reported, 24h after last exposure day)	Increased sperm DNA fragmentation, increased DNA adducts in prostate, increased clusterin expression
Dobrzynska and Radzikowska 2013	Mouse	Pzh:SFIS	Drinking water	Postnatal exposure: 8-10 weeks (14 days)	5, 10, 20, 40mg/kg	10 weeks old	Decreased sperm count and motility, increased abnormal morphology of sperm, induced sperm DNA strand breaks
El-Beshbishy et al. 2012	Rat	Albino	Oral exposure	Postnatal exposure: adult (14 days)	10mg/kg	Adults (exact age not reported, after last exposure day)	Decreased testes weight and protein content, decreased antioxidant enzymes, decreased glutathione content, increased lipid peroxidation, decreased testosterone
Fang et al 2013	Mouse	Kunming	Oral gavage	Postnatal exposure: 4-5 weeks	0.5mg/kg body weight	3 weeks after last dose	Increased oxidative stress, decreased testes weight, decreased testosterone

Source	Animal	Strain	Exposure route	Time of exposure	Doses	Age at collection	Outcome
Horstman et al. 2012	Rat	Sprague-Dawley	Subcutanous exposure	Gestational exposure: GD11-GD20	0.02, 0.5, 400mg/kg	F1: GD16, 18, or 20	Decreased *Star* expression and protein levels
Howdeshell et al. 2008	Rat	Long-Evans	Oral gavage	Gestational and postnatal exposure: GD7-PND18	2, 20, and 200µg/kg	PND150	No change in reproductive organ weight, no effect on AGD
Jin et al. 2013	Rat	Sprague-Dawley	Oral gavage	Postnatal exposure: 12-14 weeks (14 weeks)	2 µg/kg	14 weeks old	Decreased hormone production, decreased sperm and germ cell count, no change in testis weight, increased apoptosis
Kobayashi et al. 2010	Mouse	C57BL/6	Dietary exposure	Gestational, neonatal, and postnatal exposure: GD6-PND22; postnatal until sacrifice	0.05, 0.5, 5.0mg/kg	13 or 15 weeks old	Reduced sperm motility, no change in sperm count, AGD, or reproductive organ weight
Kobayashi et al. 2012	Rat	Sprague-Dawley	Dietary exposure	Gestational and neonatal exposure: GD6-PND21	0.017, 0.17, 1.7mg/kg	5 weeks or 3 months old	No change in hormones, sperm count/motility, AGD, testis, or prostate weight
LaRocca et al. 2011	Mouse	C57BL/6	Oral gavage	Gestational exposure: GD10-GD16	50 and 1000µg/kg	PND56	No effect on spermatogenesis, Sertoli cell gene expression, serum testosterone levels, or AGD
Li Y et al. 2009	Mouse	Kunming	Oral gavage	Postnatal exposure: PND31-PND44	160, 480, 960mg/kg/d	PND 45, 60, 90	Increased germ cell apoptosis, Leydig cell apoptosis, decreased spermatogenesis, increased FasL signaling
Liu et al. 2013	Rat	Wistar	Oral gavage	Postnatal exposure: 9-18 weeks old (60 days)	2, 20, 200µg/kg	18 weeks old	Decreased sperm counts, inhibited spermiation, persistent meiotic DNA strand breaks, increased germ cell apoptosis
Minamiyama et al. 2010	Rat	Wistar	Drinking water	Postnatal exposure: 8-16 weeks old (56 days)	1.0 or 10mg/L	16 weeks old	No change in sperm count, reduced sperm motility, increased production of ROS in sperm
Minamiyama et al. 2010	Rat	Wistar	Drinking water	Postnatal exposure: 12 weeks old (7 days)	1.0mg/L	12 weeks old	No change in sperm count, reduced sperm motility, increased production of ROS in sperm

Source	Animal	Strain	Exposure route	Time of exposure	Doses	Age at collection	Outcome
Nakamura et al. 2010	Rat	Wistar/st	Subcutaneous injection	Postnatal exposure: 4-9 weeks old (4 days a week for 6 weeks)	20, 100, 200mg/kg (11.4, 57.1, 114.2 mg/kg/day)	9 weeks old	Decreased serum and intratesticular testosterone, decreased steroidogenic enzyme expression, decreased *Esr1* expression, decreased Leydig cell number, decreased LH
Nanjappa et al. 2012	Rat	Long-Evans	Oral gavage	Gestational and neonatal exposure: GD12-PND21	2.5 and 25μg/kg	PND 21.35, 90	Increased Leydig cell number, decreased Leydig cell testosterone production, increased LHR, ESR1, AR
N'Tumba-Byn et al. 2012	Human	NA	*In vitro:* Fetal testes	3 days	10pM-10μM	6.5-10.5 gestational weeks	Reduced testosterone and INSL3 mRNA levels
N'Tumba-Byn et al. 2012	Rat	Wistar	*In vitro:* Fetal testes	3 days	10pM-10μM	14.5 DPC	Reduced testosterone in rodent testes
N'Tumba-Byn et al. 2012	Mouse	C57BL/6	*In vitro:* Fetal testes	3 days	10pM-10μM	12.5 DPC	Reduced testosterone in rodent testes
Okada and Kai 2008	Mouse	ICR	Silastic capsule	Gestational and neonatal exposure: Pre-breed through PND28	1.2 and 60μg/day	PND 28	Reduced mature spermatids number, no change in testosterone
Prins et al. 2011	Rat	Sprague-Dawley	Subcutaneous injection	Neonatal exposure: PND1, 3, and 5	10μg/kg	28 weeks old	Increased prostate intraepithelial neoplasia incidence
Prins et al. 2011	Rat	Sprague-Dawley	Oral exposure	Neonatal exposure: PND1, 3, and 5	10μg/kg	28 weeks old	Increased prostate intraepithelial neoplasia incidence
Qiu et al. 2013	Rat	Sprague-Dawley	Oral gavage	Postnatal exposure: 9-16 weeks old (56 days)	0.0005, 0.5, 5mg/kg	16 weeks old	Altered steroidogenic enzyme expression, decreased sperm count
Rashid et al. 2013	Mouse	Swiss albino	Oral exposure	Postnatal exposure: adult	10 mg/kg body weight	Adult, exact age unknown, after 14 days of dosing	Increased oxidative stress
Salian et al. 2009a	Rat	Holtzman	Subcutaneous injection	Neonatal exposure: PND1-PND5	100-1600μg/kg	PND15, 30, 45, 90, 125	Decreased sperm count, altered hormone production
Salian et al. 2009b	Rat	Holtzman	Oral gavage	Gestational and neonatal exposure: GD12-PND21	1.2 and 2.4μg/kg	PND125	Decreased steroid receptors in F1, F2, F3
Sánchez et al. 2013	Rat	Wistar	Subcutaneous injection	Postnatal exposure: adult (4 days)	25 and 300μg/kg	30 min after last treatment	Decreased DHT levels, no effect on testosterone levels, decreased expression of *5αR1* and *5αR2*

Source	Animal	Strain	Exposure route	Time of exposure	Doses	Age at collection	Outcome
Tainaka et al. 2012	Mouse	ICR	Subcutaneous injection	Gestational exposure: GD7 and GD14	5 and 50mg/kg	F1: 6 weeks old	Decreased sperm count, gene expression mediating spermatozoa, altered Sertoli cell number and morphology, decreased genes mediating Sertoli cell function and androgen signaling
Tang et al. 2012	Rat	Sprague-Dawley	Subcutaneous injection	Neonatal exposure: PND1, 3, and 5	10µg/kg	PND10, 90, 200	Altered methylation and expression of genes and transcriptional proteins in prostate
Thuillier et al. 2009	Rat	Sprague-Dawley	Oral gavage	Gestational exposure: GD14-PND0	0.1-200mg/kg	PND 3, 21, 60	Transient alterations in the MAPK signaling pathway
Tiwari and Vanage 2013	Rat	Holtzman	Oral gavage	Postnatal exposure: Adults (6 days)	5 and 10µg/kg	End of mating cycle (treated males; 1 to 8 weeks)	Impaired mid-spermatids and spermatocytes, decreased sperm production and motility, increased sperm DNA damage
Tyl et al. 2008	Mouse	CD-1	Dietary exposure	Gestational exposure: F0: 8 weeks pre-breed, GD1-GD14	0.003, 0.03, 0.3, 5, 50, 600mg/kg	PND1/birth	Decreased absolute AGD and delayed preputial separation, no effect on sperm, organ weight, or reproductive organ structure
Tyl et al. 2008	Mouse	CD-1	Dietary exposure	Postnatal exposure: F1: 8 weeks pre-breed	0.003, 0.03, 0.3, 5, 50, 600mg/kg	PND1/birth	Decreased absolute AGD and delayed preputial separation, no effect on sperm, organ weight, or reproductive organ structure
Wang Q et al. 2010	Mouse	CD-1	Oral gavage	Postnatal exposure: PND35-PND49	160 and 480mg/kg	PND50	Increase germ cell apoptosis, increased FasL signaling pathway, increased mitochondrial apoptotic signaling pathway
Wu et al. 2013	Rat	Sprague-Dawley	Oral gavage	Postnatal exposure: 8-10 weeks old (10 days)	200mg/kg	10 weeks old (24h after treatment)	Induced DNA damage, induced oxidative stress
Wu et al. 2011	Rat	Sprague-Dawley	Intragastric injection	Postnatal exposure: Adult (4 weeks)	10, 30, 90µg/kg	12 weeks old	Decreased testosterone

Source	Animal	Strain	Exposure route	Time of exposure	Doses	Age at collection	Outcome
Xi et al. 2011	Mouse	CD-1	Oral gavage	Gestational and neonatal exposure: F0: GD1-PND20	12, 25, 50mg/kg	PND50	Decreased steroidogenic enzyme expression, decreased testosterone
Xi et al. 2011	Mouse	CD-1	Oral gavage	Postnatal exposure: F1: PND21-PND49	12, 25, 50mg/kg	PND50	Decreased steroidogenic enzyme expression, decreased testosterone
Xi et al. 2011	Mouse	CD-1	Oral gavage	Postnatal exposure: F1: PND21-PND49	12, 25, 50mg/kg	PND50	Decreased steroidogenic enzyme expression, decreased testosterone

AGD=anogenital distance;AR=androgen receptor; DPC=days post coitum; F0=parental generation; F1=first filial generation; F2=second filial generation; F3=third filial generation; GD=gestation day; Esr1=estrogen receptor 1;Fasl=Fas ligand; INSL=insulin like factor; LH=luteinizing hormone; MAPK=mitogen activated protein kinase; PND=postnatal day; Star=steroidogenic acute regulatory protein

Table S8. BPA and sexual function outcomes in experimental studies.

Source	Animal	Strain	Exposure route	Time of exposure	Doses	Age at collection	Outcome
Adewale et al. 2009	Rat	Long-Evans	Subcutaneous injection	Neonatal exposure: PNDO-PND3	50µg/kg and 50mg/kg	PND148	Accelerated vaginal opening
DeCatanzaro et al. 2013	Mouse	CF-1	Dietary exposure	Postnatal exposure: GD10-PND9	0.175, 1.75, 17.5µg	PND60 or PND90	Increased latency to insemination, decreased intromissions with females, decreased ejaculations
DeCatanzaro et al. 2013	Mouse	CF-1	Dietary exposure	Postnatal exposure: GD10-PND9	17.5, 175, 1750µg	PND85-PND105	Increased latency to insemination, decreased intromissions with females, decreased ejaculations
Fernandez et al. 2009	Rat	Sprague-Dawley	Subcutaneous injection	Gestational exposure: PND1-10	5µg/50µL, 50µg/50µL, 500µg/50µL	PND12; adults	Accelerated puberty onset and estrous cyclicity, decreased gonadotropin releasing hormone and luteinizing hormone function
Nah et al. 2011	Mouse	ICR	Subcutaneous injection	Neonatal exposure: PND8	0.1, 1, 10, 100mg/kg	PND20-PND29; PND25, 30, 70	Accelerated vaginal onset, decreased estrous cyclicity
Ryan et al. 2010	Rat	Long-Evans	Oral gavage	Gestational and neonatal exposure: GD7-PND18	2, 20, 200µg/kg	NA	No adverse sexual function effects
Salian et al. 2009a	Rat	Holtzman	Subcutaneous injection	Neonatal exposure (males only): PND1-5	100-1600µg/kg	PND15, 30, 45, 90	Increased time taken for copulation, decreased copulation index
Salian et al. 2009b	Rat	Holtzman	Oral gavage	Gestational exposure (males only): GD12-PND21	1.2 and 2.4µg/kg	males: PND125 females: GD20	Increased time taken for copulation in F1, F2, F3

Source	Animal	Strain	Exposure route	Time of exposure	Doses	Age at collection	Outcome
Tyl et al. 2008	Mouse	CD-1	Dietary exposure	Gestational exposure: F0: 8 weeks pre-breed, GD1-GD14	0.003, 0.03, 0.3, 5, 50, 600mg/kg	PND1/birth	No adverse sexual function effects
Tyl et al. 2008	Mouse	CD-1	Dietary exposure	Postnatal exposure: F1: 8 weeks pre-breed	0.003, 0.03, 0.3, 5, 50, 600mg/kg	PND1/birth	No adverse sexual function effects

F1=first filial generation; F2=second filial generation; F3=third filial generation; GD=gestation day; PND=postnatal day

References

Adewale HB, Jefferson WN, Newbold RR, Patisaul HB. 2009. Neonatal bisphenol-A exposure alters rat reproductive development and ovarian morphology without impairing activation of gonadotropin-releasing hormone neurons. Biol Reprod 81:690-699.

Aghajanova L, Giudice LC. 2011. Effect of bisphenol A on human endometrial stromal fibroblasts *in vivo*. Reprod Biomed Online 22:249-256.

Aldad TS, Rahmani N, Leranth C, Taylor HS. 2011. Bisphenol-A exposure alters endometrial progesterone receptor expression in the nonhuman primate. Fertil Steril 96:175-179.

An BS, Ahn HJ, Kang HS, Jung EM, Yang H, Hong EJ, Jeung EB. 2013. Effects of estrogen and estrogenic compounds, 4-tert-octylphenol, and bisphenol A on the uterine contraction and contraction-associated proteins in rats. Mol Cell Endocrinol 375:27-34.

Anjum S, Rahman S, Kaur M, Ahmad F, Rashid H, Ansari Ra, Raisuddin S. 2011. Melatonin ameliorates bisphenol A-induced biochemical toxicity in testicular mitochondria of mouse. Food Chem Toxicol 49:2849-54.

Arase S, Ishii K, Igarashi K, Aisaki K, Yoshio Y, Matsushima A, et al. 2011. Endocrine disrupter bisphenol A increases in situ estrogen production in the mouse urogenital sinus. Biol Reprod 84(4):734-42.

Benachour N, Aris A. 2009. Toxic effects of low doses of Bisphenol-A on human placental cells. Toxicol Appl Pharmacol 241:322-328.

Berger RG, Shaw J, deCatanzaro D. 2008. Impact of acute bisphenol-A exposure upon intrauterine implantation of fertilized ova and urinary levels of progesterone and 17beta-estradiol. Reprod Toxicol 26:94-99.

Berger RG, Foster WG, DeCatanzaro D. 2010. Bisphenol-A exposure during the period of blastocyst implantation alters uterine morphology and perturbs measures of estrogen and progesterone receptor expression in mice. Reprod Toxicol 30:393-400.

Bloom MS, Kim D, vom Saal FS, Taylor JA, Cheng G, Lamb JD et al. 2011a. Bisphenol A exposure reduces the estradiol response to gonadotropin stimulation during *in vivo* fertilization. Fertil Steril 96:672-677.

Bloom MS, vom Saal FS, Kim D, Taylor JA, Lamb JD, Fujimoto VY. 2011b. Serum unconjugated bisphenol A concentrations in men may influence embryo quality indicators during *in vivo* fertilization. Environ Toxicol Pharmacol 32:319-323.

Bosquiazzo VL, Varayoud J, Munoz-de-Toro M, Luque EH, Ramos JG. 2010. Effects of neonatal exposure to bisphenol A on steroid regulation of vascular endothelial growth factor expression and endothelial cell proliferation in the adult rat uterus. Biol Reprod 82:86-95.

Bredhult C, Sahlin L, Olovsson M. 2009. Gene expression analysis of human endometrial endothelial cells exposed to Bisphenol A. Reprod Toxicol 28:18-25.

Brieño-Enriquez MA, Robles P, Camats-Tarruella N, Garcia-Cruz R, Roig I, Cabero L et al. 2011a. Human meiotic progression and recombination are affected by bisphenol A exposure during *in vivo* human oocyte development. Hum Reprod 26:2807-2818.

Brieño-Enriquez MA, Reig-Viader R, Cabero L, Toran N, Martinez F, Roig I, et al. 2011b. Gene expression is altered after bisphenol A exposure in human fetal oocytes *in vivo*. Mol Hum Reprod 2012; 18:171-183.

Bromer JG, Zhou Y, Taylor MB, Doherty L, Taylor HS. 2010. Bisphenol-A exposure in utero leads to epigenetic alterations in the developmental programming of uterine estrogen response. FASEB J 2010; 24:2273-2280.

Buck Louis GM, Peterson CM, Chen Z, Croughan M, Sundaram R, Stanford J et al. 2013. Bisphenol A and phthalates and endometriosis: the Endometriosis: Natural History, Diagnosis and Outcomes Study. Fertil Steril 100:162-9.

Cabaton NJ, Wadia PR, Rubin BS, Zalko D, Schaeberle CM, Askenase MH, et al. 2011. Perinatal exposure to environmentally relevant levels of bisphenol A decreases fertility and fecundity in CD-1 mice. Environ Health Perspect 119:547-552.

Cantonwine D, Meeker JD, Hu H, Sanchez BN, Lamadrid-Figueroa H, Mercado-Garcia A, et al. 2010. Bisphenol A exposure in Mexico City and risk of prematurity: a pilot nested case control study. Environ Health 9:62.

Castro B, Sanchez P, Torres JM, Preda O, del Moral RG, Ortega E. 2013. Bisphenol A exposure during adulthood alters expression of aromatase and 5alpha-reductase isozymes in rat prostate. PLoS One 8(2):e55905.

Chao HH, Zhang XF, Chen B, Pan B, Zhang LJ, Li L, et al. 2012. Bisphenol A exposure modifies methylation of imprinted genes in mouse oocytes via the estrogen receptor signaling pathway. Histochem Cell Biol 137:249-259.

Chitra KC, Latchoumycandane C, Mathur PP. 2003. Induction of oxidative stress by bisphenol A in the epididymal sperm of rats. Toxicology 185:119-27.

Chou WC, Chen JL, Lin CF, Chen YC, Shih FC, Chuang CY. 2011. Biomonitoring of bisphenol A concentrations in maternal and umbilical cord blood in regard to birth outcomes and adipokine expression: a birth cohort study in Taiwan. Environ Health 10:94.

Cobellis L, Colacurci N, Trabucco E, Carpentiero C, Grumetto L. 2009. Measurement of bisphenol A and bisphenol B levels in human blood sera from healthy and endometriotic women. Biomed. Chromatogr 2009; 23:1186-1190.

D'Cruz SC, Jubendradass R, Jayakanthan M, Rani SJ, Mathur PP. 2012a. Bisphenol A impairs insulin signaling and glucose homeostasis and decreases steroidogenesis in rat testis: an *in vivo* and in silico study. Food Chem Toxicol 50(3-4):1124-33.

D'Cruz SC, Jubendradass R, Mathur PP. 2012b. Bisphenol A induces oxidative stress and decreases levels of insulin receptor substrate 2 and glucose transporter 8 in rat testis. Reprod Sci 19:163-172.

DeCatanzaro D, Berger RG, Guzzo AC, Thorpe JB, Khan A. 2013. Perturbation of male sexual behavior in mice (Mus musculus) within a discrete range of perinatal bisphenol-A doses in the context of a high- or low-phytoestrogen diet. Food Chem Toxicol 55:164-171.

De Flora S, Micale RT, La Maestra S, Izzotti A, D'Agostini F, Camoirano A, et al. 2011. Upregulation of clusterin in prostate and DNA damage in spermatozoa from bisphenol A-treated rats and formation of DNA adducts in cultured human prostatic cells. Toxicol Sci 122:45-51.

Dobrzynska MM, Radzikowska J. 2013. Genotoxicity and reproductive toxicity of bisphenol A and X-ray/bisphenol A combination in male mice. Drug Chem Toxicol 36:19-26.

Ehrlich S, Williams PL, Missmer SA, Flaws JA, Ye X, Calafat AM et al. 2012a. Urinary bisphenol A concentrations and early reproductive health outcomes among women undergoing IVF. Hum. Reprod 27:3583-3592.

Ehrlich S, Williams PL, Missmer SA, Flaws JA, Berry KF, Calafat AM, et al. 2012b. Urinary Bisphenol A concentrations and implantation failure among women undergoing in vitro fertilization. Environ Health Perspect 120(7):978-83.

Ehrlich S, Williams PL, Hauser R, Missmer SA, Peretz J, Calafat AM, Flaws JA. 2013. Urinary bisphenol A concentrations and cytochrome P450 19 A1 (*Cyp19*) gene expression in ovarian granulosa cells: an *in vivo* human study. Reprod Toxicol 42:18-23.

Eichenlaub-Ritter U, Vogt E, Cukurcam S, Sun F, Pacchierotti F, Parry J. 2008. Exposure of mouse oocytes to bisphenol A causes meiotic arrest but not aneuploidy. Mutat Res 651:82-92.

El Beshbishy HA, Aly HA, El Shafey M. 2012. Lipoic acid mitigates bisphenol A-induced testicular mitochondrial toxicity in rats. Toxicol Ind. Health DOI: 10.1177/0748233712446728.

Environmental Protection Agency (EPA). Integrated Risk Information Systems: Bisphenol A (CASRN 80-05-7). Updated 07/01/1993. http://www.epa.gov/iris/subst/0356.htm#bib.

Fang Y, Zhou Y, Zhong Y, Gao X, Tan T. 2013. Effect of vitamin E on reproductive functions and anti-oxidant activity of adolescent mamel mice exposed to bisphenol A. Wei Sheng Yan Jiu 42:18-22.

Fenichel P, Dechaux H, Harthe C, Gal J, Ferrari P, Pacini P, et al. 2012. Unconjugated bisphenol A cord blood levels in boys with descended or undescended testes. Hum Reprod 27:983-990.

Fernandez M, Bianchi M, Lux-Lantos V, Libertun C. 2009. Neonatal exposure to bisphenol A alters reproductive parameters and gonadotropin releasing hormone signaling in female rats. Environ Health Perspect 117:757-762.

Fernandez M, Bourguignon N, Lux-Lantos V, Libertun C. 2010. Neonatal exposure to bisphenol A and reproductive and endocrine alterations resembling the polycystic ovarian syndrome in adult rats. Environ Health Perspect 118:1217-1222.

Fujimoto VY, Kim D, vom Saal FS, Lamb JD, Taylor JA, Bloom MS. 2011. Serum unconjugated bisphenol A concentrations in women may adversely influence oocyte quality during *in vivo* fertilization. Fertil Steril 95:1816-1819.

Galloway T, Cipelli R, Guralnik J, Ferrucci L, Bandinelli S, Corsi AM, et al. 2010. Daily bisphenol A excretion and associations with sex hormone concentrations: results from the InCHIANTI adult population study. Environ Health Perspect 118:1603-1608.

Gayrard V, Lacroix MZ, Collet SH, Viguie C, Bousquet-Melou A, Toutain PL, Picard-Hagen N. 2013. High bioavailability of bisphenol A from sublingual exposure. Environ Health Perpect 121:951-6.

Grasselli F, Baratta L, Baioni L, Bussolati S, Ramoni R, Grolli S, et al. 2010. Bisphenol A disrupts granulosa cell function. Domest. Anim Endocrinol 39:34-39.

Hampton T. 2013. NIH panel: Name change, new priorities advised for polycystic ovary syndrome. JAMA 309:863.

Hiyama M, Choi EK, Wakitani S, Tachibana T, Khan H, Kusakabe KT, et al. 2011. Bisphenol-A (BPA) affects reproductive formation across generations in mice. J Vet Med Sci 73:1211-1215.

Horstman KA, Naciff JM, Overmann GJ, Foertsch LM, Richardson BD, Daston GP. 2012. Effects of transplacental 17-α-ethynyl estradiol or bisphenol A on the developmental profile of steroidogenic acute regulatory protein in the rat testis. Birth Defects Res B Dev Reprod Toxicol 95(4):318-25.

Howdeshell KL, Furr J, Lambright CR, Wilson VS, Ryan BC, Gray LE, Jr. 2008. Gestational and lactational exposure to ethinyl estradiol, but not bisphenol A, decreases androgen-dependent reproductive organ weights and epididymal sperm abundance in the male Long Evans hooded rat. Toxicol Sci 102:371-382.

Hunt PA, Koehler KE, Susiarjo M, Hodges CA, Illagan A, Voigt RC, et al. 2003. Bisphenol A exposures causes meiotic aneuploidy in the female mouse. Curr Biol 13:546-53.

Hunt PA, Susiarjo M, Rubio C, Hassold TJ. 2009. The bisphenol A experience: a primer for the analysis of environmental effects on mammalian reproduction. Biol Reprod 81(5):807-13.

Hunt PA, Lawson C, Gieske M, Murdoch B, Smith H, Marre A, et al. 2012. Bisphenol A alters early oogenesis and follicle formation in the fetal ovary of the rhesus monkey. Proc Natl Acad Sci U. S. A . 109:17525-17530.

Iguchi T, Fukazawa Y, Uesugi Y, Taksugi N. 1990. Polyovular follicles in mouse ovaries exposed neonatally to diethylstibestrolin vivoandin vitro. Biol Reprod 43 478–484.

Jin P, Wang X, Chang F, Bai Y, Li Y, Zhou R, Chen L. 2013. Low dose bisphenol A impairs spermatogenesis by suppressing reproductive hormone production and promoting germ cell apoptosis in adult rats. J Biomed Res 27:135-144.

Kabuto H, Hasuike S, Minagawa N, Shishibori T. 2003. Effects of bisphenol A on the metabolisms of active oxygen species in mouse tissues. Environ Res 93:31-5.

Kabuto H, Amakawa M, Shishibori T. 2004. Exposure to bisphenol A during embryonic/fetal life and infancy increases oxidative injury and cause underdevelopment of the brain and testes in mice. Life Sci 30:2931-40.

Kandaraki E, Chatzigeorgiou A, Livadas S, Palioura E, Economou F, Koutsilieris M, et al. 2011. Endocrine disruptors and polycystic ovary syndrome (PCOS): elevated serum levels of bisphenol A in women with PCOS. J Clin Endocrinol Metab 96:E480-E484.

Kobayashi K, Kubota H, Ohtani K, Hojo R, Miyagawa M. 2012. Lack of effects for dietary exposure of bisphenol A during in utero and lactational periods on reproductive development in rat offspring. J Toxicol Sci 37:565-573.

Kobayashi K, Ohtani K, Kubota H, Miyagawa M. 2010. Dietary exposure to low doses of bisphenol A: effects on reproduction and development in two generations of C57BL/6J mice. Congenit. Anom. (Kyoto) 50:159-170.

LaRocca J, Boyajian A, Brown C, Smith SD, Hixon M. 2011. Effects of in utero exposure to Bisphenol A or diethylstilbestrol on the adult male reproductive system. Birth Defects Res B Dev. Reprod Toxicol 92:526-533.

Lawson C, Gieske M, Murdoch B, Ye P, Li Y, Hassold T, et al. 2011. Gene expression in the fetal mouse ovary is altered by exposure to low doses of bisphenol A. Biol Reprod 84:79-86.

Lee BE, Park H, Hong YC, Ha M, Kim Y, Chang N, Kim BN, Kim YJ, Yu SD, Ha EH. 2013. Prenatal bisphenol A and birth outcomes: MOCEH (Mothers and Children's Environmental Health) study. Int J Hyg Environ Health. pii: S1438-4639(13)00099-0. doi: 10.1016/j.ijheh.2013.07.005.

Lee SG, Kim JY, Chung JY, Kim YJ, Park JE, Oh S, Yoon YD, Yoo KS, Yoo YH, Kim JM. 2013. Bisphenol A exposure during adulthood causes augmentation of follicular atresia and luteal regression by decreasing 17beta-estradiol synthesis via downregulation of aromatase in rat ovary. Environ Health Perspect 121:663-669.

Lee SH, Kang SM, Choi MH, Lee J, Park MJ, Kim SH, Lee WY, Hong J, Chung BC. 2013. Changes in steroid metabolism among girls with precocious puberty may not be associated with urinary levels of bisphenol A. Reprod Toxicol. doi:10.1016/j.reprotox.2013.03.008.

Lenie S, Cortvrindt R, Eichenlaub-Ritter U, Smitz J. 2008. Continuous exposure to bisphenol A during *in vivo* follicular development induces meiotic abnormalities. Mutat Res 651:71-81.

Li DK, Zhou Z, Qing D, He Y, Wu T, Miao M, et al. 2010. Occupational exposure to bisphenol-A (BPA) and the risk of self-reported male sexual dysfunction. Hum. Reprod 25:519-527.

Li DK, Zhou Z, Miao M, He Y, Wang J, Ferber J, et al. 2011. Urine bisphenol-A (BPA) level in relation to semen quality. Fertil Steril 95:625-630.

Li Y, Zhang W, Liu J, Wang W, Li H, Zhu J, Weng S, Xiao S, Wu T. 2013. Prepubertal bisphenol A exposure interferes with ovarian follicle development and its relevant gene expression. Reprod Toxicol. pii: S0890-6238(13)00348-1. doi:10.1016/j.reprotox.2013.09.002.

Li YJ, Song TB, Cai YY, Zhou JS, Song X, Zhao X, et al. 2009. Bisphenol A exposure induces apoptosis and upregulation of Fas/FasL and caspase-3 expression in the testes of mice. Toxicol Sci 108(2):427-36.

Liu C, Duan W, Li R, Xu S, Zhang L, Chen C, He M, Lu Y, Wu H, Pi H, Luo X, Zhang Y, Zhong M, Yu Z, Zhou Z. 2013. Exposure to bisphenol A disrupts meiotic progression during spermatogenesis in adult rats through estrogen-like activity. Cell Death Dis 4:e676.

Machtinger R, Combelles CM, Missmer SA, Correia KF, Williams P, Hauser R, Racowsky C. 2013. Bisphenol-A and human oocyte maturation *in vitro*. Hum Reprod 28:2735-45.

Meeker JD, Calafat AM, Hauser R. 2010a. Urinary bisphenol A concentrations in relation to serum thyroid and reproductive hormone levels in men from an infertility clinic. Environ Sci Technol 44:1458-1463.

Meeker JD, Ehrlich S, Toth TL, Wright DL, Calafat AM, Trisini AT, et al. 2010b. Semen quality and sperm DNA damage in relation to urinary bisphenol A among men from an infertility clinic. Reprod Toxicol 30:532-539.

Mendiola J, Jorgensen N, Andersson AM, Calafat AM, Ye X, Redmon JB et al. 2010. Are environmental levels of bisphenol A associated with reproductive function in fertile men? Environ Health Perspect 118:1286-1291.

Mendoza-Rodriguez CA, Garcia-Guzman M, Baranda-Avila N, Morimoto S, Perrot-Applanat M, Cerbon M. 2011. Administration of bisphenol A to dams during perinatal period modifies molecular and morphological reproductive parameters of the offspring. Reprod Toxicol 31:177-183.

Miao M, Yuan W, He Y, Zhou Z, Wang J, Gao E, et al. 2011a. In utero exposure to bisphenol-A and anogenital distance of male offspring. Birth Defects Res A Clin Mol Teratol 91:867-872.

Miao M, Yuan W, Zhu G, He X, Li DK. 2011b. In utero exposure to bisphenol-A and its effect on birth weight of offspring. Reprod Toxicol 32:64-68.

Minamiyama Y, Ichikawa H, Takemura S, Kusunoki H, Naito Y, Yoshikawa T. 2010. Generation of reactive oxygen species in sperms of rats as an earlier marker for evaluating the toxicity of endocrine-disrupting chemicals. Free Radic Res 44(12):1398-406.

Mok-Lin E, Ehrlich S, Williams PL, Petrozza J, Wright DL, Calafat AM, et al. 2010. Urinary bisphenol A concentrations and ovarian response among women undergoing IVF. Int J Androl 33:385-393.

Morice L, Benaitreau D, Dieudonne MN, Morvan C, Serazin V, de Mazancourt P, et al. 2011. Antiproliferative and proapoptotic effects of bisphenol A on human trophoblastic JEG-3 cells. Reprod Toxicol 32:69-76.

Muhlhauser A, Susiarjo M, Rubio C, Griswold J, Gorence G, Hassold T, et al. 2009. Bisphenol A Effects on the Growing Mouse Oocyte Are Influenced by Diet. Biol Reprod 80:1067–1072. DOI: 10.1095/biolreprod.108.074815

Nah WH, Park MJ, Gye MC. 2011. Effects of early prepubertal exposure to bisphenol A on the onset of puberty, ovarian weights, and estrous cycle in female mice. Clin Exp Reprod Med 38:75-81.

Nakamura D, Yanagiba Y, Duan Z, Ito Y, Okamura A, Asaeda N, et al. 2010. Bisphenol A may cause testosterone reduction by adversely affecting both testis and pituitary systems similar to estradiol. Toxicol Lett 194:16-25.

Nanjappa MK, Simon L, Akingbemi BT. 2012. The industrial chemical bisphenol A (BPA) interferes with proliferative activity and development of steroidogenic capacity in rat Leydig cells. Biol Reprod 86(5):135,1-12.

Newbold RR, Jefferson WN, Padilla-Banks E. 2009. Prenatal exposure to bisphenol a at environmentally relevant doses adversely affects the murine female reproductive tract later in life. Environ Health Perspect 117:879-885.

Nicholson TM, Ricke EA, Marker PC, Miano JM, Mayer RD, Timms BG, et al. 2012. Testosterone and 17beta-estradiol induce glandular prostatic growth, bladder outlet obstruction, and voiding dysfunction in male mice. Endocrinology 153(11):5556-65.

Nicholson TM, Ricke WA. 2011. Androgens and estrogens in benign prostatic hyperplasia: past, present and future. Differentiation 82(4-5):184-99.

N'Tumba-Byn T, Moison D, Lacroix M, Lecureuil C, Lesage L, Prud'homme SM, Pozzi-Gaudin S, Frydman R, Benachi A, Livera G, Rouiller-Fabre V, Habert R. 2013. Differential effects of bisphenol A and diethylstilbestrol on human, rat and mouse fetal Leydig cell function. PLoS One. 7(12):e51579. doi: 10.1371/journal.pone.0051579.

Okada A, Kai O. 2008. Effects of estradiol-17beta and bisphenol A administered chronically to mice throughout pregnancy and lactation on the male pups' reproductive system. Asian J Androl 10:271-276.

Pacchierotti F, Ranaldi R, Eichenlaub-Ritter U, Attia S, Adler ID. 2008. Evaluation of aneugenic effects of bisphenol A in somatic and germ cells of the mouse. Mutat Res 651:64-70.

Padmanabhan V, Siefert K, Ransom S, Johnson T, Pinkerton J, Anderson L, et al. 2008. Maternal bisphenol-A levels at delivery: a looming problem? J Perinatol 28:258-263.

Peretz J, Gupta RK, Singh J, Hernandez-Ochoa I, Flaws JA. 2011. Bisphenol A impairs follicle growth, inhibits steroidogenesis, and downregulates rate-limiting enzymes in the estradiol biosynthesis pathway. Toxicol Sci 119:209-217.

Peretz J, Craig ZR, Flaws JA. 2012. Bisphenol A inhibits follicle growth and induces atresia in cultured mouse antral follicles independently of the genomic estrogenic pathway. Biol Reprod 87(3):63.

Philippat C, Mortamais M, Chevrier C, Petit C, Calafat AM, Ye X, Silva MJ, Brambilla C, Pin I, Charles MA, Cordier S, Slama R. 2012. Exposure to phthalates and phenols during pregnancy and offspring size at birth. Environ Health Perspect. 120(3):464-70

Prins GS, Ye SH, Birch L, Ho SM, Kannan K. 2011. Serum bisphenol A pharmacokinetics and prostate neoplastic responses following oral and subcutaneous exposures in neonatal Sprague-Dawley rats. Reprod Toxicol 31:1-9.

Qiao L, Zheng L, Cai D. 2010. Study on the levels of the bisphenol A, octylphenol, 4-nonylphenol in serum of precocious girls. Wei Sheng Yan Jiu 39(1):9-12.

Qiu LL, Wang X, Zhang XH, Zhang Z, Gu J, Liu L, Wang Y, Wang X, Wang SL. 2013. Decreased androgen receptor expression may contribute to spermatogenesis failure in rats exposed to low concentration of bisphenol A. Toxicol Lett 219:116-124.

Rashid H, Ahmad F, Rahman S, Ansari Ra, Bhatia K, Kaur M, Islam F, Raisuddin S. 2009. Iron deficient augments bisphenol A-induced oxidative stress in rats. Toxicology 256:7-12.

Richter CA, Birnbaum LS, Farabollini F, Newbold RR, Rubin BS, Talsness CE, Vandenbergh JG, Walser-Kuntz DR, vom Saal FS. 2007. *In vivo* effects of bisphenol A in laboratory rodent studies. Reprod Toxicol. 24(2):199-224.

Ricke WA, McPherson SJ, Bianco JJ, Cunha GR, Wang Y, Risbridger GP. 2008. Prostatic hormonal carcinogenesis is mediated by in situ estrogen production and estrogen receptor alpha signaling. FASEB J 22(5):1512-20.

Rivera OE, Varayoud J, Rodriguez HA, Munoz-de-Toro M, Luque EH. 2011. Neonatal exposure to bisphenol A or diethylstilbestrol alters the ovarian follicular dynamics in the lamb. Reprod Toxicol 32:304-312.

Rodriguez HA, Santambrosio N, Santamaria CG, Munoz-de-Toro M, Luque EH. 2010. Neonatal exposure to bisphenol A reduces the pool of primordial follicles in the rat ovary. Reprod Toxicol 2010; 30:550-557.

Ross C, Morriss A, Khairy M, Khalaf Y, Braude P, Coomarasamy A, et al. 2010. A systematic review of the effect of oral antioxidants on male infertility. Reprod Biomed Online 20(6):711-23.

Ryan BC, Hotchkiss AK, Crofton KM, Gray LE, Jr. 2010. In utero and lactational exposure to bisphenol A, in contrast to ethinyl estradiol, does not alter sexually dimorphic behavior, puberty, fertility, and anatomy of female LE rats. Toxicol Sci 114:133-148.

Salian S, Doshi T, Vanage G. 2009a. Neonatal exposure of male rats to bisphenol A impairs fertility and expression of Sertoli cell junctional proteins in the testis. Toxicology 265:56-67.

Salian S, Doshi T, Vanage G. 2009b. Perinatal exposure of rats to bisphenol A affects the fertility of male offspring. Life Sci 85:742-752.

Sánchez P, Castro B, Torres JM, Olmo A, Del Moral RG, Ortega E. 2013. Bisphenol A modifies the regulation exerted by testosterone on 5 α -reductase isozymes in ventral prostate of adult rats. Biomed Res Int. 2013:629235. doi:10.1155/2013/629235.

Signorile PG, Spugnini EP, Mita L, Mellone P, D'Avino A, Bianco M, et al. 2010. Pre-natal exposure of mice to bisphenol A elicits an endometriosis-like phenotype in female offspring. Gen Comp Endocrinol 168:318-325.

Snijder CA, Heederik D, Pierik FH, Hofman A, Jaddoe VW, Koch HM, Longnecker MP, Burdorf A. 2013. Fetal growth and prenatal exposure to bisphenol A: The generation R study. Environ Health Perspect. 121(3):393-8. doi:10.1289/ehp.1205296.

Susiarjo M, Hassold TJ, Freeman E, Hunt PA. 2007. Bisphenol A exposure in utero disrupts early oogenesis in the mouse. PLoS Genet 12;3(1):e5.

Susiarjo M, Sasson I, Mesaros C, Bartolomei MS. 2013. Bisphenol A exposure disrupts genomic imprinting in the mouse. PLoS Genet 9(4):e1003401. doi:10.1371/journal.pgen.1003401.

Tainaka H, Takahashi H, Umezawa M, Tanaka H, Nishimune Y, Oshio S, et al. 2012. Evaluation of the testicular toxicity of prenatal exposure to bisphenol A based on microarray analysis combined with MeSH annotation. J Toxicol Sci 37:539-548.

Tan W, Huang H, Wang Y, Wong TY, Wang CC, Leung LK. 2013. Bisphenol A differentially activates protein kinase C isoforms in murine placental tissue. Toxicol Appl Pharmacol. 269(2):163-8. doi:10.1016/j.taap.2013.03.016.

Tang WY, Morey LM, Cheung YY, Birch L, Prins GS, Ho SM. 2012. Neonatal exposure to estradiol/bisphenol A alters promoter methylation and expression of Nsbp1 and Hpcal1 genes and transcriptional programs of Dnmt3a/b and Mbd2/4 in the rat prostate gland throughout life. Endocrinology 153(1):42-55.

Thuillier R, Manku G, Wang Y, Culty M. 2009. Changes in MAPK pathway in neonatal and adult testis following fetal estrogen exposure and effects on rat testicular cells. Microsc Res Tech 72:773-786.

Tiwari D, Vanage G. 2013. Mutagenic effect of Bisphenol A on adult rat male germ cells and their fertility. Reprod Toxicol.40:60-8. doi: 10.1016/j.reprotox.2013.05.013.

Trapphoff T, Heiligentag M, El Hajj N, Haaf T, Eichenlaub-Ritter U. 2013. Chronic exposure to a low concentration of bisphenol A during follicle culture affects the epigenetic status of germinal vesicles and metaphase II oocytes. Fertil Steril. pii: S0015-0282(13)02968-3. doi: 10.1016/j.fertnstert.2013.08.021.

Tyl RW, Myers CB, Marr MC, Sloan CS, Castillo NP, Veselica MM, et al. 2008. Two-generation reproductive toxicity study of dietary bisphenol A in CD-1 (Swiss) mice. Toxicol Sci 104:362-384.

Vandenberg LN, Chahoud I, Heindel JJ. Padmanabhan V, Paumgartten FJ, Schoenfelder G. 2010. Urinary, circulating, and tissue biomonitoring studies indicate widespread exposure to bisphenol A. Environ Health Perspect 118:1055-70.

Vandenberg LN, Ehrlich S, Belcher SM, Ben-Jonathan N, Dolinoy Dc, Hugo ER, Hunt PA, Rubin BS, Saili KS, Soto AM, Wang H-S, vom Saal FS. 2013. Low dose effects of bisphenol A: An integrated review of *in vitro*, laboratory animal, and epidemiology studies. Endocrine Disruptors 1:1e25078.

Varayoud J, Ramos JG, Bosquiazzo VL, Lower M, Munoz-de-Toro M, Luque EH. 2011. Neonatal exposure to bisphenol A alters rat uterine implantation-associated gene expression and reduces the number of implantation sites. Endocrinology 152:1101-1111.

Varayoud J, Ramos JG, Bosquiazzo VL, Munoz-de-Toro M, Luque EH. 2008. Developmental exposure to bisphenol A impairs the uterine response to ovarian steroids in the adult. Endocrinology 149:5848-5860.

Veiga-Lopez A, Luense LJ, Christenson LK, Padmanabhan V. 2013. Developmental programming: gestational bisphenol-A treatment alters trajectory of fetal ovarian gene expression. Endocrinology. 154(5):1873-84.

vom Saal FS, Akingbemi BT, Belcher SM, Birnbaum LS, Crain DA, Eriksen M, et al. 2007. Chapel Hill bisphenol A expert panel consensus statement: integration of mechanisms, effects in animals and potential to impact human health at current levels of exposure. Reprod Toxicol 24:131-138.

vom Saal FS, VandeVoort C, Taylor J, Welshons W, Toutain P-L, Hunt PA. 2014. Bisphenol A (BPA) pharmacokinetics with daily oral bolus or continuous exposure via silastic capsules in pregnant rehesus monkeys:relevance for human exposures. Reprod Toxicol, in press. http://dx.doi.org/10.1016/j.reprotox.2014.01.007

Wang Q, Zhao XF, Ji YL, Wang H, Liu P, Zhang C, et al. 2010. Mitochondrial signaling pathway is also involved in bisphenol A induced germ cell apoptosis in testes. Toxicol Lett 199(2):129-35.

Wang Z, Liu BC, Lin GT, Lin CS, Lue TF, Willingham E, et al. 2007. Up-regulation of estrogen responsive genes in hypospadias: microarray analysis. J Urol 177(5):1939-46.

Willingham E, Baskin LS. 2007. Candidate genes and their response to environmental agents in the etiology of hypospadias. Nat Clin Pract Urol 4(5):270-9.

Wolff MS, Britton JA, Boguski L, Hochman S, Maloney N, Serra N, Liu Z, Berkowitz G, Larson S, Forman J. 2008a. Environmental exposures and puberty in inner-city girls. Environ Res 107(3):393-400.

Wolff MS, Engel SM, Berkowitz GS, Ye X, Silva MJ, Zhu C, Wetmur J, Calafat AM. 2008b. Prenatal phenol and phthalate exposures and birth outcomes. Environ Health Perspect. 116(8):1092-7.

Wolff MS, Teitelbaum SL, Pinney SM, Windham G, Liao L, Biro F, Kushi LH, Erdmann C, Hiatt RA, Rybak ME, Calafat AM; Breast Cancer and Environment Research Centers. 2010. Investigation of relationships between urinary biomarkers of phytoestrogens, phthalates, and phenols and pubertal stages in girls. Environ Health Perspect. 118(7):1039-46.

Wu HJ, Liu C, Duan WX, Xu SC, He MD, Chen CH, Wang Y, Zhou Z, Yu ZP, Zhang L, Chen Y. 2013. Melatonin ameliorates bisphenol A-induced DNA damage in the germ cells of adult male rats. Mutat Res 752:57-67.

Wu JH, Jiang XR, Liu GM, Liu XY, He GL, Sun ZY. 2011. Oral exposure to low-dose bisphenol A aggravates testosterone-induced benign hyperplasia prostate in rats. Toxicol Ind Health 27:810-819.

Xi W, Lee CK, Yeung WS, Giesy JP, Wong MH, Zhang X, et al. 2011. Effect of perinatal and postnatal bisphenol A exposure to the regulatory circuits at the hypothalamus-pituitary-gonadal axis of CD-1 mice. Reprod Toxicol 31:409-417.

Xiao S, Diao H, Smith MA, Song X, Ye X. 2011. Preimplantation exposure to bisphenol A (BPA) affects embryo transport, preimplantation embryo development, and uterine receptivity in mice. Reprod Toxicol 32:434-441.

Yigit F, Daglioglu S. 2010. Histological changes in the uterus of the hens after embryonic exposure to bisphenol A and diethylstilbestrol. Protoplasma 247:57-63.

Zhang HQ, Zhang XF, Zhang LJ, Chao HH, Pan B, Feng YM, et al. 2012. Fetal exposure to bisphenol A affects the primordial follicle formation by inhibiting the meiotic progression of oocytes. Mol Biol Rep 39:5651-5657.

Zhang XF, Zhang LJ, Feng YN, Chen B, Feng YM, Liang GJ, et al. 2012. Bisphenol A exposure modifies DNA methylation of imprint genes in mouse fetal germ cells. Mol Biol Rep 39:8621-8628.

Zhou W, Liu J, Liao L, Han S, Liu J. 2008. Effect of bisphenol A on steroid hormone production in rat ovarian theca-interstitial and granulosa cells. Mol Cell Endocrinol 283:12-18.

Ziv-Gal A, Craig ZR, Wang W, Flaws JA. 2013. Bisphenol A inhibits cultured mouse ovarian follicle growth partially via the aryl hydrocarbon receptor signaling pathway. Reprod Toxicol. 42C:58-67. doi:10.1016/j.reprotox.2013.07.022.
